# Elevated Lactate by High-Intensity Interval Training Regulates the Hippocampal BDNF Expression and the Mitochondrial Quality Control System

**DOI:** 10.3389/fphys.2021.629914

**Published:** 2021-02-25

**Authors:** Jingyun Hu, Ming Cai, Qinghui Shang, Zhaorun Li, Yu Feng, Beibei Liu, Xiangli Xue, Shujie Lou

**Affiliations:** ^1^Key Laboratory of Exercise and Health Sciences of Ministry of Education, Shanghai University of Sport, Shanghai, China; ^2^College of Rehabilitation Sciences, Shanghai University of Medicine & Health Sciences, Shanghai, China; ^3^Clinical Medicine Department, Weifang Medical University, Weifang, China

**Keywords:** High-intensity interval training, lactate, mitochondrial quality control system, BDNF, mouse hippocampus

## Abstract

High-intensity interval training (HIIT) is reported to be beneficial to brain-derived neurotrophic factor (BDNF) biosynthesis. A key element in this may be the existence of lactate, the most obvious metabolic product of exercise. *In vivo*, this study investigated the effects of a 6-week HIIT on the peripheral and central lactate changes, mitochondrial quality control system, mitochondrial function and BDNF expression in mouse hippocampus. *In vitro*, primary cultured mice hippocampal cells were used to investigate the role and the underlying mechanisms of lactate in promoting mitochondrial function during HIIT. *In vivo* studies, we firstly reported that HIIT can potentiate mitochondrial function [boost some of the mitochondrial oxidative phosphorylation (OXPHOS) genes expression and ATP production], stimulate BDNF expression in mouse hippocampus along with regulating the mitochondrial quality control system in terms of promoting mitochondrial fusion and biogenesis, and suppressing mitochondrial fission. In parallel to this, the peripheral and central lactate levels elevated immediately after the training. *In vitro* study, our results revealed that lactate was in charge of regulating mitochondrial quality control system for mitochondrial function and thus may contribute to BDNF expression. In conclusion, our study provided the mitochondrial mechanisms of HIIT enhancing brain function, and that lactate itself can mediate the HIIT effect on mitochondrial quality control system in the hippocampus.

## Introduction

High-intensity interval training (HIIT) is a type of exercise involving several sets of alternating epochs of high intensity [shorter periods of workloads near or above peak aerobic capacity (VO_2__max_)] and low intensity (longer periods of rest or about 40–60% HR_max_ exercise) (Haram et al., 2009; Gibala et al., 2012; Nokia et al., 2016; Robinson et al., 2018). This pattern of exercise is more effective than conventional aerobic exercise in promoting brain health, such as cognitive function enhancement (Afzalpour et al., 2015; Saucedo Marquez et al., 2015; Nokia et al., 2016; Slusher et al., 2018; Luan et al., 2019). Both clinical and animal experiments show that HIIT can evoke higher levels of brain-derived neurotrophic factor (BDNF) production to improve the brain function in health, diabetics, and stroke patients (Afzalpour et al., 2015; Tonoli et al., 2015; Saucedo Marquez et al., 2015; Di Battista et al., 2018; Freitas et al., 2018; Boyne et al., 2019). However, it is still unclear how HIIT promotes BDNF production. One of the important underlying reason is likely to be that HIIT efficiently regulates mitochondrial quality control system for mitochondrial function enhancement, which is beneficial to improving hippocampal BDNF expression. Evidence for this is that 12-week HIIT can reverse cognitive impairment in APP/PS1 transgenic mice via promoting hippocampal mitochondrial fusion and simultaneously inhibiting mitochondrial fission (Li et al., 2019).

Mitochondrial function is mainly regulated by the mitochondrial quality control system, which includes mitochondrial fusion, fission, mitophagy, and biogenesis. Mitochondrial fusion is regulated by mitofusin 1 (MFN1), mitofusin 2 (MFN2), and optic atrophy (OPA1). MFN1 and MFN2 regulate outer mitochondrial membrane (OMM) fusion, while OPA1 orchestrates inner mitochondrial membrane fusion. These proteins act together to repair the dysfunctional mitochondria by mixing their contents with those of healthy mitochondria (Rovira-Llopis et al., 2017). Mitochondrial fission will then segregate the badly damaged mitochondria. This process is mainly regulated by cytosolic GTPase dynamin-related protein1 (DRP1) and mitochondrial fission 1 protein (FIS1) in mammals (Cai and Tammineni, 2016). DRP1 can assemble into a ring-like structure that squeezes cellular membranes to divide mitochondria into two parts. FIS1 is anchored to the OMM, and its function is to recruit DRP1 from the cytoplasm to the OMM (Flippo and Strack, 2017). In this process, the mitophagy programme is initiated to eliminate bad mitochondria, and the mitochondrial biogenesis signal is activated to renew mitochondrial production (Tatsuta and Langer, 2008; Vina et al., 2009; Bernardo et al., 2016; Zhang et al., 2016). In mitophagy, damaged mitochondria can be recognized by autophagosomes to trigger their degradation and packaging into lysosomes, which is regulated by the PTEN-induced putative kinase 1 (PINK1)-PARKIN signaling pathway (Yoon et al., 2011). Activated PINK1 recruits PARKIN for polyubiquitination, known as cytosolic E3-ubiquitin ligase. The polyubiquitinated substrates are then recognized by microtubule-associated proteins light chain 3 (LC3) adapters, such as Sequestosome-1 (P62), to interact with LC3 and recruit the tagged mitochondria to autophagosomes (Yoo and Jung, 2018).

For mitochondrial biogenesis, peroxisome proliferator-activated receptor γ coactivator-1α (PGC-1α) is the first stimulator, and it acts as a key factor connecting several regulator cascades (Fernandez-Marcos and Auwerx, 2011; Gureev et al., 2019). It activates the nuclear respiratory factors NRF1 and NRF2. Subsequently, they activate the expression of transcription factor A (TFAM), and as a result, they increase the transcription and translation of mitochondrial DNA (mtDNA). This in turn leads to duplication of mitochondria. Briefly, these mitochondrial pathways ensure the continuous production of new mitochondria in the cell and the timely removal of old or damaged mitochondria from the cellular compartment. Therefore, the mitochondrial quality control system is essential for the maintenance of mitochondrial function, such as oxidative phosphorylation (OXPHOS) related-genes and ATP. However, to date, it is unclear how HIIT influences the hippocampal mitochondrial quality control system and mitochondrial function.

Recently, the diverse roles of lactate in mediating brain function have been brought into focus, and the positive effect of exercise on brain function has been demonstrated to be mediated by lactate (Schiffer et al., 2011; Tsukamoto et al., 2016a, b; Charalambous et al., 2018; Hashimoto et al., 2018; Malik et al., 2018; Rodriguez et al., 2018; Boyne et al., 2019). During HIIT, blood lactate levels can increase from baseline (∼2 mM in human and ∼3 mM in mouse) to 10∼15 mM in human and mouse (Morland et al., 2017; Coxon et al., 2018). The high levels of blood lactate then cross the blood-brain-barrier (BBB) via monocarboxylic acid transporter1 (MCT1) into the brain (Bergersen, 2015). Moreover, lactate can also be produced via glycolysis in astrocytes and transported by monocarboxylic acid transporter 4 (MCT4) and MCT1 into the extracellular space (Salmina et al., 2015; Takashi Matsuia et al., 2017). As a result, the level of brain lactate obviously increases (Rasmussen et al., 2011; Wiegers et al., 2017). The increased lactate can potentiate neuronal synaptic plasticity to promote learning and memory (Schurr et al., 1988; Yang et al., 2014; Margineanu et al., 2018; El Hayek et al., 2019; Scavuzzo et al., 2020), and control neuronal activity to mediate brain excitability (Bozzo et al., 2013; Tang et al., 2014; Herrera-Lopez and Galvan, 2018; de Castro Abrantes et al., 2019). In addition, lactate is likely to participate in the improvement and maintenance of brain mitochondrial function. *In vitro* studies show that ATP production for supplying neuronal energy depends on lactate (Shen et al., 2015; Vardjan et al., 2018), and lactate can also promote mitochondrial biogenesis (Hashimoto et al., 2007; Lezi et al., 2013).

Therefore, we speculate that increased hippocampal lactate immediately after HIIT may enhance the hippocampal mitochondrial function and promote BDNF expression via regulating mitochondrial quality control system. To test this hypothesis, we use both vivo and vitro study and identify a hitherto-unknown role of lactate. We highlight that HIIT, as a non-invasive stimulating manner, represents a promising strategy to boost brain fitness. Furthermore, ingesting 10∼20 mM lactate to replace strenuous exercise (above the lactate threshold), at a safe and effective physiological level, maybe a great strategy for people in urgent need of ameliorating brain function incapable of exercise.

## Materials and Methods

### Animals

Male mice were 7 weeks of age at the start of the experiments and procured from Nanjing Model Animal Research Center (Certificate SCXK 2018-0008). They were housed in cages and provided with food and water *ad libitum*. The room temperature was maintained at (22–24)°C under a 12 h light-dark cycle. After 1 week acclimatization period, mice were divided randomly into two groups (*n* = 22 each group): sedentary control group (Ctl group) and HIIT group. Mice were anesthetized with diethyl to sacrifice within 1 h (for the hippocampal lactate detection) or 24 h (for the other indicators detection) after 6-week exercise paradigm. The hippocampus was dissected on crushed ice immediately and then stored at −80°C for later analysis. Animal care and use were in accordance with the guidelines set by the Institutional Animal Ethics Committee (IAEC) and as approved by the Ethical Committee for Science Research at Shanghai (102772019DW010).

### HIIT Paradigm

**FIGURE 1:**
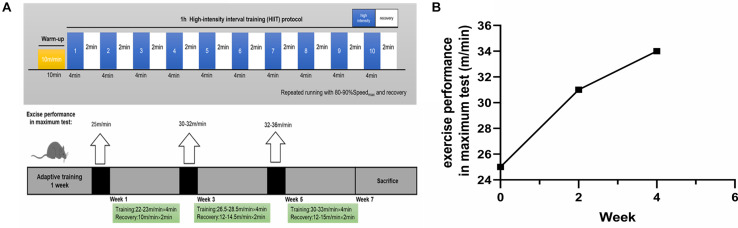
The exercise paradigm of 6-week HIIT and exercise performance in the maximum test. **(A)** The exercise paradigm of 6-week HIIT. In the first and second week, the HIIT group completed warm-up (10 min × 10 m/min) and then 10 bouts of high-intensity running [4 min × (22–23) m/min], and separated by active rest (2 min × 10 m/min). In the third and fourth week, the HIIT group completed warm-up and then 10 bouts of high-intensity running [4 min × (26.5–28.5) m/min], and separated by active rest [2 min × (12–14.5) m/min]. In the fifth and sixth week, the HIIT group completed warm-up and then 10 bouts of high-intensity running [4 min × (30–33) m/min], and separated by active rest [2 min × (12–15) m/min]. **(B)** The exercise performance in the maximum test. The exercise performance of the HIIT group was, respectively, performed at the end of adaptive training, the second and the fourth week of training.

Mice in the HIIT group were familiarized with five bouts of treadmill running (4 min × (20–22) m/min) and active rest (2 min × 10 m/min) for 5 days. After familiarization, the mice completed 6-week HIIT. They were exposed to the HIIT exercise protocol for five consecutive days each week. This exercise regime has been previously described (Morland et al., 2017). Briefly, each session consisted of warm-up (10 min × 10 m/min), followed by 10 bouts of 4 min high-intensity running (4 min × (80–90)% Speed_max_), and separated by active rest (2 min × (40–50)% Speed_max_). Running took place on a treadmill at a 0 degree (Figure 1A). The maximal speed test for mice was, respectively, performed at the end of adaptive training, the second and the fourth week of training, and adjust the speed of interval training (Figure 1B). In this regard, the beginning speed of mice was 10 m/min for lasting 10 min to warm up. Then the speed was increased by 2 m/min every 2 min until exhaustion. Throughout the experiment, the Ctl group did not receive any exercise training.

### Primary Hippocampal Cell Culture

Primary hippocampal cells were obtained from 1-day-old neonatal C56BL/6J mice as previously described (Cai et al., 2020), which were purchased from Shanghai JSJ Lab Animal Center (Certificate SCXK 2013-0016, Shanghai, China). Briefly, the dissociated hippocampal tissues were collected in ice-cold D-Hanks’ balanced salt solution (HBSS; No. H6648, Sigma-Aldrich, United States) and digested into a single cell suspension in 0.25% trypsin/EDTA (No. 25200072, Gibco, United States) for 10–15 min. Termination of the digestion is supplemented with Dulbecco’s Modified Eagle Medium (DMEM; No. 11960044, Gibco, United States), which contained 10% Fetal Bovine Serum (No. 10099141, Gibco, United States). Primary hippocampal cells (1.0 × 10^6^ cells/ml) were maintained with serum-free Neurobasal Medium (No. 10888022, Gibco, United States) containing 2% B27 (No. 17504044, Gibco, United States) and 1% penicillin-streptomycin (No. C0222, Beyotime, Shanghai, China) in 6-well plates, which were previously coated with 0.01% poly-L-lysine (No. P4832, Sigma-Aldrich, United States). Hippocampal cells were cultured in a humidified incubator at 37°C with 5% CO_2_ with a half- medium changed every 2 days. After 7 days, the cells were harvested for the follow-up experiments.

### Cell Treatment

On 5th day, cells were treated with different concentrations (5, 10, 15, and 20 mM) of sodium L- lactate (No. L7022, Sigma-Aldrich, United States) for 3, 6, 12, and 24 h at the incubator. Lactate was prepared as 20 mM stock in configured Neurobasal medium before using.

### Lactate Measurements

Blood lactate was detected by Lactate-Scout (EKF Co., Germany) immediately at the end of 2nd, 4th, and 6th week after completing the 10 bouts of training. Ten of all mice in each group were collected from tail tip blood. Hippocampal lactate level was measured using the method described by El Hayek et al. (2019). At the end of the 6-week training and after the anesthetization, 20 mice (*n* = 10 each group) were sacrificed immediately. Hippocampal lactate levels were measured using the L-lactate assay kit (No. ab65331, Abcam) according to the manufacturer’s protocol. We used TCA Kit (No. ab204708, Abcam) to remove enzyme from tissue sample in case of endogenous LDH degrading lactate.

### Western Blotting

At the end of the 6-week training, 24 h after the last training and after the anesthetization, all mice were sacrificed. The left hippocampus of each mouse brain was carefully dissected out following the standard procedure. The hippocampus of each mice (six animals per group randomly) was washed with pre-cooling PBS. The sample was homogenated at 14,000 g for 5 min in RIPA Lysis Buffer (No. P0013B, Beyotime, Shanghai, China), which contained phosphatase and protease inhibitors, with 1 mM PMSF (No. ST506, Beyotime, Shanghai, China), and the supernatant was collected. Material from vitro experiments was processed similarly. Proteins (30 μg) in SDS were loaded on a 10–12.5% polyacrylamide gradient gel. The gel was then transferred to 0.22 μm PVDF membrane (Epizyme Biotech, Shanghai, China). The membranes were blocked with Protein Free Rapid Blocking Buffer (No. PS108P Epizyme Biotech, Shanghai, China) for 10 min and then incubated for 10 h at 4°C with primary antibodies. After washing, the membranes were incubated for 1 h with secondary antibodies. Quantification of the band density was performed using the ImageJ. Protein levels were normalized to the band intensity of TUBULIN. Details of the antibodies used are provided below (Table 1).

**TABLE 1:** Antibodies used for WB analysis of mice proteins.

Antibody name	Molecular weight (kDa)	Classification	Antibody source	Catalog number	Dilution ratio
MFN1	84	mitochondrial fusion	Proteintech	13798-1-AP	1:2000
MFN2	80		CST	9482	1:1000
OPA1	80–100		CST	80471	1:1000
DRP1	82	mitochondrial fission	CST	8570S	1:1000
FIS1	17		Proteintech	10956-1-AP	1:1000
PINK1	66	mitochondrial autophagy	Abcam	ab23707	1:1000
PARKIN	50		CST	4211	1:1000
P62	62		MBI	PM045	1:1000
LC3II/I	16, 14		CST	12741	1:1000
PGC-1α	91	mitochondrial biogenesis	Novus	NBP1-04676	1:1000
NRF1	68		CST	46743	1:1000
NRF2	97		CST	12721	1:1000
TFAM	28		Abcam	ab131607	1:2000
MCT1	54	monocarboxylic acid transporter	Abcam	ab90582	1:1000
MCT4	49		Proteintech	22787-1-AP	1:1000
B -TUBULIN	50		Proteintech	10068-1-AP	1:2000

### RNA Extraction and Real-Time PCR (RT-PCR)

Total RNA was prepared from right hippocampal tissues or primary cells using TRIZOL (Ambion, United States). The RNA quality was assessed on a NanoDrop 2000 (Thermo, United States), where the 260/280 ratio was obtained. Samples with a ratio of 1.8–2.0 were processed for gene analysis. Reverse transcription was performed using PrimeScript^TM^RT Master Mix (No. RR036A, Takara) according to the manufacturer’s protocol. Real-time PCR was performed using SYBR^®^ Premix Ex Taq II (No. RR820A, Takara) and StepOnePlus Real-Time PCR System (Applied Biosystems, CA, United States). The mRNA levels were normalized to the internal loading control of GAPDH and determined with the comparative ΔΔCT method. OXPHOS related genes include NDUFS8, SDHb, Uqcrc1, COX5b, and Atp5a1. Details of the Primers used are provided below (Table 2).

**TABLE 2:** Primer sequences used for RT-PCR analysis of mice cDNA.

Gene name	Primer	Sequence (5′–3′)
NDUFS8	Forward	AGTGGCGGCAACGTACAAG
	Reverse	TCGAAAGAGGTAACTTAGGGTCA
SDHb	Forward	AATTTGCCATTTACCGATGGGA
	Reverse	AGCATCCAACACCATAGGTCC
Uqcrc1	Forward	ACGCAAGTGCTACTTCGCA
	Reverse	CAGCGTCAATCCACACTCCC
COX5b	Forward	TCTAGTCCCGTCCATCAGCAAC
	Reverse	GCAGCCAAAACCAGATGACAGT
Atp5a1	Forward	TCTCCATGCCTCTAACACTCG
	Reverse	CCAGGTCAACAGACGTGTCAG
MCT1	Forward	TGTTAGTCGGAGCCTTCATTTC
	Reverse	CACTGGTCGTTGCACTGAATA
MCT4	Forward	TCACGGGTTTCTCCTACGC
	Reverse	GCCAAAGCGGTTCACACAC
GAPDH	Forward	AGGTCGGTGTGAACGGATTTG
	Reverse	TGTAGACCATGTAGTTGAGGTCA

### gDNA Extraction and RT-PCR

gDNA was prepared from left hippocampal tissues or primary cells using Takara MiniBEST Universal Genomic DNA Extraction Kit (No. 9765, Takara) according to the manufacturer’s instructions. The DNA quality was assessed on a NanoDrop 2000 (Thermo), where the 260/280 ratio was obtained. Samples with a ratio of 1.8–2.0 were processed for gene analysis. RT-PCR were performed using SYBR^®^ Premix Ex Taq II (No. RR820A, Takara). The mitochondrial DNA copy number was determined by RT-PCR using primers specific for cytochrome B (mt-Cytb)-a mitochondrial genome encoded gene and was normalized to levels of a nuclear-encoded gene cytochrome C (Cycs). Relative mitochondrial DNA levels between groups were quantified by comparative ΔΔCT method as previously described (Guo et al., 2009; Fanibunda et al., 2019). Details of the Primers used are provided below (Table 3).

**TABLE 3:** Primer sequences used for mtDNA analysis of mice.

Gene name	Primer	Sequence (5′–3′)
mt-Cytb	Forward	TGCATACGCCATTCTACG
	Reverse	ATGGGTGTTCTACTGGTTG
Cycs	Forward	CAGTGCAGAATTACCAGGTGTG
	Reverse	GGTCTGCCCTTTCTCCCTTCT

### ATP Detection

The ATP levels in the right hippocampus and primary cells were measured using the method described by Sheng et al. (2009) according to the manufacturer’s instruction (No. S0026, Beyotime, Shanghai, China). Briefly, the hippocampal tissue or harvested cells were lysed with ATP lysis buffer and then centrifugated at 12,000 *g* for 5 min at 4°C. A total of 20 μl supernatant was mixed with 100 μl luciferase reagent in a lighttight microplate, which was measured by Luminance. ATP levels were normalized to the protein content of each sample, estimated using a BCA protein assay kit (No. P0010, Beyotime, Shanghai, China) and expressed as fold change of treated over control.

### Statistics

All data are presented as means ± SEM. *In vivo* study, comparisons of two groups were performed using an unpaired Student’s *t* test. *In vitro* study, comparisons of two groups were performed using non-parametric Mann-Whitney. Comparisons of multiple groups were performed using non-parametric Kruskal-Wallis *H* test. A *p* value of 0.05 or less was considered statistically significant. All calculations and the graph construction were performed using SPSS 20.0 and GraphPad 8.0 software (La Jolla, CA, United States).

## Results

### HIIT Increased Lactate Levels and Promoted Hippocampal MCT1/4 and BDNF Expression

**FIGURE 2:**
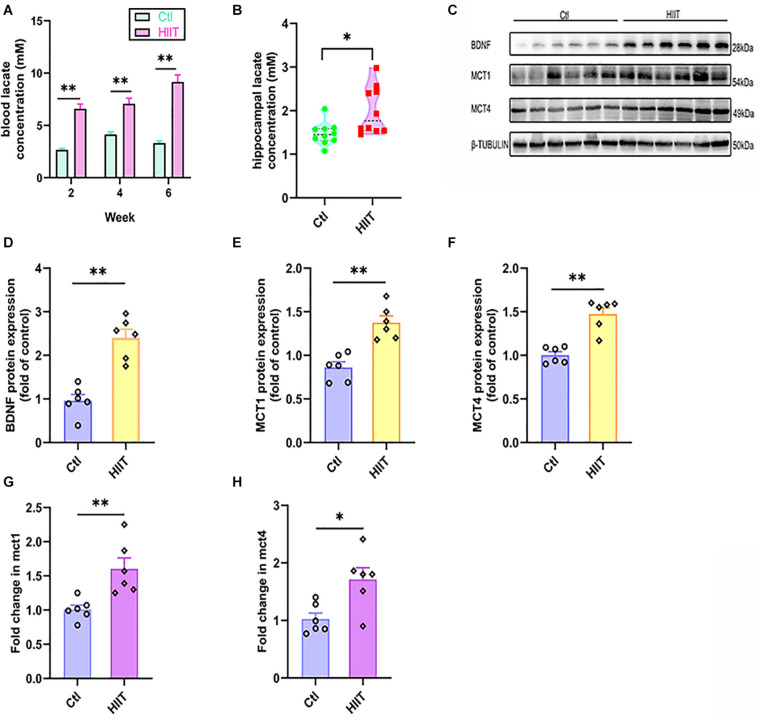
HIIT increased lactate levels and promoted hippocampal MCT1/4 and BDNF expression. The blood lactate levels in two groups were detected at the end of 2nd, 4th, and 6th week of training to estimate the exercise intensity of High-intensity interval training (HIIT). The hippocampal lactate level was determined by colorimetry. The levels of Monocarboxylic acid transporter 1 (MCT1) and Monocarboxylic acid transporter 4 (MCT4) were also detected by RT-PCR and WB, respectively, to indirectly evaluate the lactate level in mouse hippocampus. The protein expression of Brain-derived neurotrophic factor (BDNF) was detected by WB. **(A)** The blood lactate levels. **(B)** hippocampal lactate level. **(C)** Representative WB image. **(D)** BDNF protein expression. **(E)** MCT1 protein expression. **(F)** MCT4 protein expression. **(G)** mct1 mRNA level. **(H)** mct4 mRNA level. TUBULIN and gapdh as the loading control. *N* = 10 mice per group for **(A,B)**. *N* = 6 mice per group for **(D-H)**. ^∗^*p* ≤ 0.05 and ^∗∗^*p* ≤ 0.01 as compared with the Ctl group by unpaired Student’s *t* test. Values are expressed as mean ± standard error of the mean.

The blood lactate was detected at the end of 2nd, 4th, and 6th week of training to estimate the exercise intensity. We found that the blood lactate levels in HIIT group can reach to 6∼10 mM immediately after training at the end week of 2nd (6.6 ± 0.44 mM in the HIIT group vs. 2.67 ± 0.14 mM in the Ctl group, *t*(10.81) = −8.43, *p* < 0.01), 4th (7.07 ± 0.54 mM in the HIIT group vs. 4.13 ± 0.25 mM in the Ctl group, *t*(18) = −4.89, *p* < 0.01) and 6th (9.15 ± 0.68 mM in the HIIT group vs. 3.32 ± 0.20 mM in the Ctl group, *t*(18) = −8.21, *p* < 0.01), which are significantly increased compared with the Ctl group, respectively (Figure 2A). The hippocampal lactate levels in HIIT group were also significantly higher than those in the Ctl group (1.90 ± 0.52 mM in the HIIT group vs. 1.39 ± 0.25 mM in the Ctl group, *t*(12.91) = −2.81, *p* = 0.02, Figure 2B), the results suggested that this high-intensity exercise regime can effectively increase the lactate levels. Moreover, HIIT promoted mRNA and protein expression of MCT1 and MCT4 in mouse hippocampus (*t*(10) = −5.14, *p* < 0.01 for MCT1 protein; *t*(10) = −3.50, *p* = 0.01 for MCT1 mRNA; *t*(10) = −5.86, *p* < 0.01 for MCT4 protein; *t*(10) = −3.02, *p* = 0.01 for MCT4 mRNA, Figures 2C,E–H), suggesting that the blood lactate and astrocytic glycogen-derived lactate were transported into intercellular space for hippocampal lactate increase. Meanwhile, the mice hippocampal BDNF expression was higher in the HIIT group than the Ctl group (*t*(10) = −6.102, *p* < 0.01 Figures 2C,D).

### The Effects of HIIT on Hippocampal Mitochondrial Function

**FIGURE 3:**
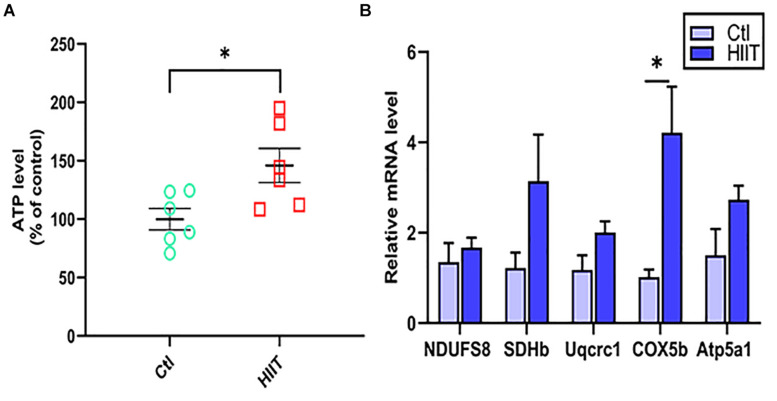
The effects of HIIT on hippocampal mitochondrial function. The level of ATP was detected by Luciferase and the oxidative phosphorylation (OXPHOS) related genes (NDUFS8, SDHb, Uqcrc1, COX5b, and Atp5a1) was detected by RT-PCR in mouse hippocampus. **(A)** The ATP level. **(B)** OXPHOS mRNA levels. The gapdh as the loading control. *N* = 6 mice per group. ^∗^*P* ≤ 0.05 as compared with the Ctl group by unpaired Student’s *t* test. Values are expressed as mean ± standard error of the mean.

The levels of hippocampal mitochondrial OXPHOS related genes (NDUFS8, SDHb, Uqcrc1, COX5b, Atp5a1) expression and ATP were used to reflect mitochondrial function. The results showed that HIIT significantly promoted COX5b gene expression (*t*(5.27) = −3.08, *p* = 0.03, Figure 3B), but there was no significant difference in the gene expression of NDUFS8, SDHb, Uqcrc1, Atp5a1. Meanwhile, we found that HIIT notably elevated hippocampal ATP levels (*t*(10) = −2.67, *p* = 0.02, Figure 3A). The above results demonstrated that HIIT had the potential to enhance hippocampal mitochondrial function.

### The Effects of HIIT on Hippocampal Mitochondrial Fusion and Fission

**FIGURE 4:**
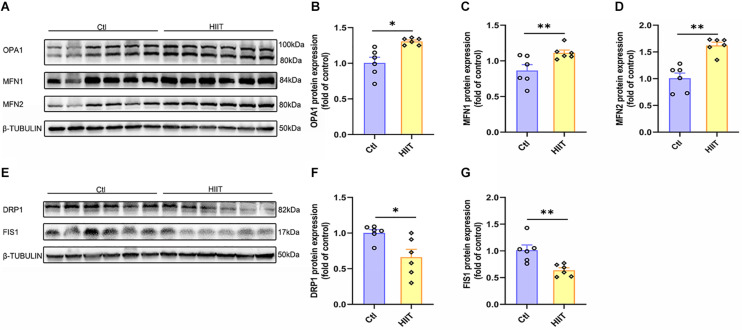
The effects of HIIT on hippocampal mitochondrial fusion and fission. The Optic atrophy (OPA1), Mitofusin 1 (MFN1), and Mitofusin 2 (MFN2) were determined by WB to represent the mitochondrial fusion state in mouse hippocampus. The Dynamin-related protein1 (DRP1) and mitochondrial fission 1 protein (FIS1) were also determined by WB to represent the mitochondrial fission state in mouse hippocampus. **(A,E)** Representative WB image. **(B)** OPA1 protein expression. **(C)** MFN1 protein expression. **(D)** MFN2 protein expression. **(F)** DRP1 protein expression. **(G)** FIS1 protein expression. TUBULIN as the loading control. *N* = 6 mice per group. ^∗^*p* ≤ 0.05 and ^∗∗^*p* ≤ 0.01 as compared with the Ctl group by unpaired Student’s *t* test. Values are expressed as mean ± standard error of the mean.

Mitochondrial function is mainly influenced by the mitochondrial quality control system, fusion and fission are of important components. The results showed that HIIT promoted the fusion protein expression of OPA1, MFN1, and MFN2 (*t*(5.64) = −3.75, *p* = 0.01; *t*(7.13) = −2.74, *p* = 0.03; *t*(10) = −5.38, *p* < 0.01, Figures 4E–G), but significantly inhibited the fission protein expression of DRP1 and FIS1 (*t*(6.64) = 2.87, *p* = 0.03; *t*(10) = −3.56, *p* = 0.01, Figures 4A–D). These results demonstrated that the significant increase in the above oxidative phosphorylation levels in hippocampus may be due to the enhanced mitochondrial fusion during the HIIT.

### The Effects of HIIT on Hippocampal Mitophagy

**FIGURE 5:**
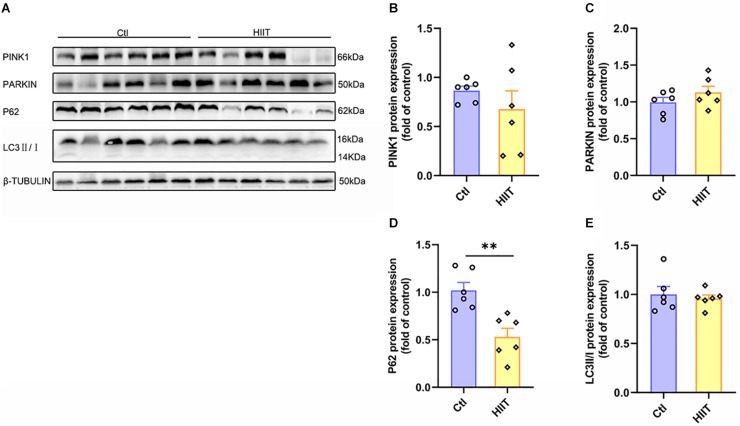
The effects of HIIT on hippocampal mitophagy. The PTEN-induced putative kinase 1 (PINK1), PARKIN, Sequestosome-1 (P62), and microtubule-associated protein light chain3 (LC3) were determined by WB to represent the mitophagy state in mouse hippocampus. **(A)** Representative WB image. **(B)** PINK1 protein expression. **(C)** PARKIN protein expression. **(D)** P62 protein expression. **(E)** LC3 protein expression. TUBULIN as the loading control. *N* = 6 mice per group. ^∗∗^*P* ≤ 0.01 as compared with the Ctl group by unpaired Student’s *t* test. Values are expressed as mean ± standard error of the mean.

As an acute tissue stress response occurred, mitochondrial fusion and fission are often accompanied with mitophagy. The PINK1 mediated mitophagy signals can be activated to remove the damaged mitochondria. Our results showed that there was no significant difference between the two groups in protein expression of PINK1, PARKIN, and LC3II/I (Figures 5A–C,E), although the protein expression of P62 was decreased in HIIT group compared to Ctl group (*t*(10) = 3.97, *p* < 0.01, Figures 5A,D). The results suggested that HIIT had little effect on mice hippocampal mitophagy, namely, HIIT didn’t damage hippocampal mitochondria.

### The Effects of HIIT on Hippocampal Mitochondrial Biogenesis Signal and Mitochondria DNA Copy Number

**FIGURE 6:**
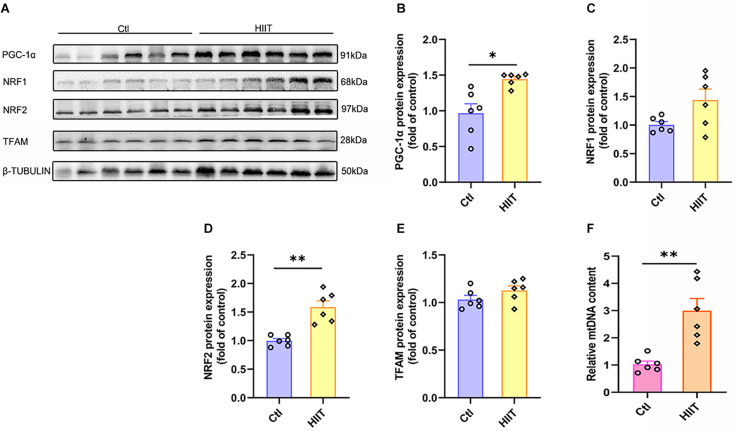
The effects of HIIT on hippocampal mitochondrial biogenesis signal and mitochondria DNA copy number. The peroxisome proliferator-activated receptor γ coactivator-1α (PGC-1α), nuclear respiratory factors 1 (NRF1), nuclear respiratory factors 1 (NRF2), and transcription factor A (TFAM) were determined by WB to evaluate the mitochondrial biogenesis signals in mouse hippocampus. The mt-Cytb level was determined by RT-PCR to evaluate the mitochondria DNA copy number. **(A)** Representative WB image. **(B)** PGC-1α protein expression. **(C)** NRF1 protein expression. **(D)** NRF2 protein expression. **(E)** TFAM protein expression. **(F)** mt-Cytb level. TUBULIN and Cycs as the loading control. *N* = 6 mice per group. ^∗^*p* ≤ 0.05 and ^∗∗^*p* ≤ 0.01 as compared with the Ctl group by unpaired Students *t* test. Values are expressed as mean ± standard error of the mean.

Mitochondrial biogenesis is critical for generating new mitochondria and maintaining mitochondrial homeostasis, which is orchestrated by PGC-1α-regulated signaling pathway. PGC-1α activates TFAM by interacting with NRF1 and NRF2, causing mitochondrial DNA replication and transcription. We found HIIT promoted hippocampal PGC-1α and NRF2 protein expression (*t*(5.78) = −3.49, *p* = 0.01; *t*(6.17) = −5.13, *p* < 0.01, Figures 6A,B,D), accompanied by the mitochondrial copy number increased significantly (*t*(5.66) = −4.24, *p* = 0.01, Figure 6F). However, the protein expression of NRF1 and TFAM had no significant difference (Figures 6A,C,E). These results demonstrated that HIIT could facilitate hippocampal mitochondrial biogenesis signal and increase mitochondrial copy number to accommodate high energy requirements.

### Lactate Increased the OXPHOS Related Genes Expression and ATP Levels in Primary Hippocampal Cells

**FIGURE 7:**
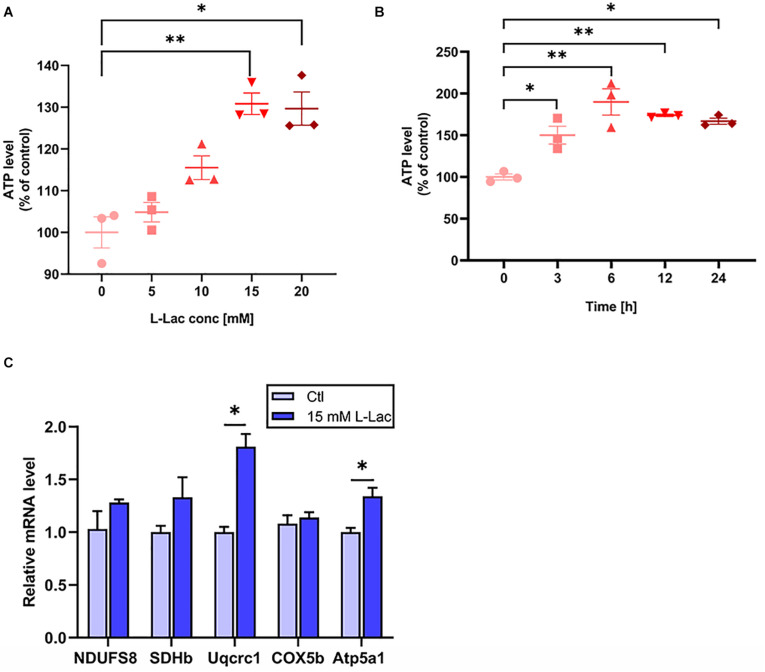
Lactate increased the OXPHOS related genes expression and ATP levels in primary hippocampal cells. The mice primary cultured hippocampal cells were treated with different concentration of L-lactate (5, 10, 15, and 20 mM) for 24 h and different time points (3, 6, 12, and 24 h) following 15 mM lactate treatment for determination of the effect of lactate on the levels of ATP. The levels of ATP were detected by Luciferase. The oxidative phosphorylation (OXPHOS) related genes (NDUFS8, SDHb, Uqcrc1, COX5b, and Atp5a1) was detected in the condition of 15 mM L-lactate treatment for 3 h by RT-PCR in primary hippocampal cells. **(A,B)** The ATP levels. **(C)** OXPHOS mRNA levels. The gapdh as the loading control. *N* = 3 in three independent experiments. ^∗^*P* ≤ 0.05 and ^∗∗^*P* ≤ 0.01 as compared with the Ctl group by non-parametric tests. Values are expressed as mean ± standard error of the mean.

To assess whether lactate, a metabolite produced by HIIT, directly regulates mice hippocampal mitochondrial function, we adopted primary mice hippocampal cell culture with lactate treatment *in vitro* and detected OXPHOS subunits expression and ATP levels. We firstly detected the ATP levels with different concentrations of lactate (5, 10, 15, and 20 mM) for 24 h. The ATP levels were evoked by lactate stimulus from 15 to 20 mM (*H* = 12.43, *p* = 0.01, Figure 7A). The 15 mM (*p* = 0.01) and 20 mM (*p* = 0.02) lactate treatment are significantly to the 0 mM lactate treatment. Then we detected the cellular ATP level with 15 mM lactate for different time points (0, 3, 6, 12, and 24 h) (*H* = 9.83, *p* = 0.04 Figure 7B). The ATP levels were increased as early as 3 h and reached to the peak at 6 h after lactate treatment (*p* = 0.05, *p* = 0.01, Figure 7B). Based on this, we adopted the 15 mM lactate treatment for 3 h in the subsequent experiment. Then we found the gene levels of Uqcrc1 and Atp5a1 (representing complex III and ATP synthase, respectively) significantly increased after lactate treatment (*Z* = −1.96, *p* = 0.05; *Z* = −1.99, *p* = 0.046, Figure 7C), and there was an increased tendency in the levels of the gene NDUFS8, SDHb, and COX5b. The results confirmed that a high concentration of lactate could partly enhance the mitochondrial ability of OXPHOS related genes expression and augment ATP levels in the primary hippocampal cells.

### Lactate Increased BDNF Expression in Primary Hippocampal Cells

**FIGURE 8:**
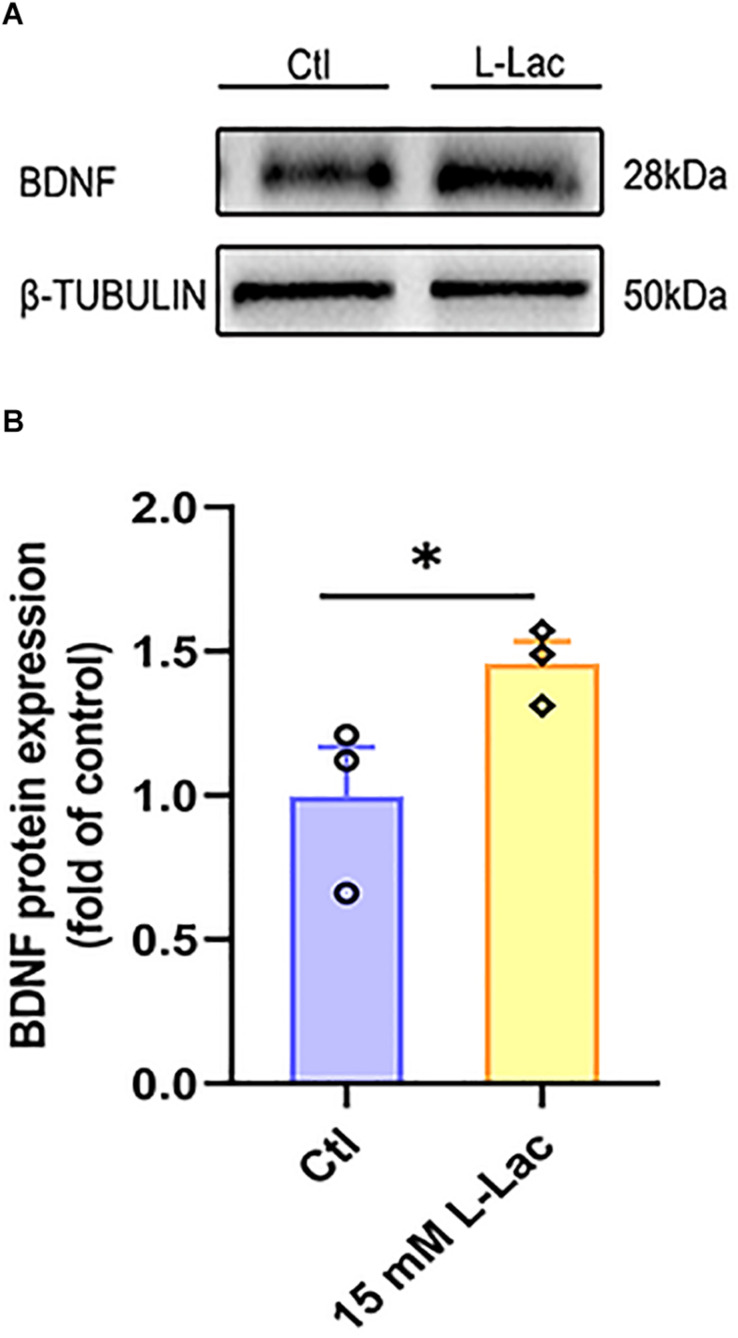
Lactate increased BDNF expression in primary hippocampal cells. The mice primary cultured hippocampal cells were treated with 15 mM L-lactate for 3 h to observe the effect of lactate on BDNF expression. The protein expression of Brain-derived neurotrophic factor (BDNF) was detected by WB. **(A)** Representative WB image. **(B)** BDNF protein expression. TUBULIN as the loading control. *N* = 3 in three independent experiments. ^∗^*P* ≤ 0.05 compared with the Ctl group by non-parametric tests. Values are expressed as mean ± standard error of the mean.

In order to understand the role of lactate in HIIT promoting mice hippocampal BDNF expression, we detected the protein expression of BDNF in the condition of lactate treatment in mice hippocampal cells. Our result showed that the BDNF protein expression increased in the mice hippocampal cells following the lactate treatment (*Z* = −1.96, *p* = 0.05, Figures 8A,B).

### Lactate Directly Regulated Mitochondrial Fusion and Fission in Primary Hippocampal Cells

**FIGURE 9:**
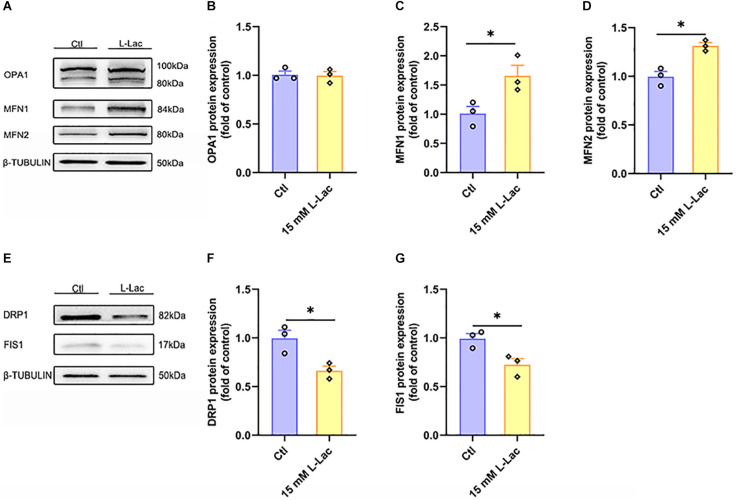
Lactate directly regulated mitochondrial fusion and fission in primary hippocampal cells. The Optic atrophy (OPA1), Mitofusin 1 (MFN1), and Mitofusin 2 (MFN2) were determined by WB to observe the effect of lactate on the mitochondrial fusion in primary hippocampal cells. The Dynamin-related protein1 (DRP1) and mitochondrial fission 1 protein (FIS1) were also determined by WB to observe the effect of lactate on the mitochondrial fusion in primary hippocampal cells. **(A,E)** Representative WB image. **(B)** OPA1 protein expression. **(C)** MFN1 protein expression. **(D)** MFN2 protein expression. **(F)** DRP1 protein expression. **(G)** FIS1 protein expression. TUBULIN as the loading control. *N* = 3 in three independent experiments. ^∗^*P* ≤ 0.05 as compared with the Ctl group by non-parametric tests. Values are expressed as mean ± standard error of the mean.

To further determine the mechanism of lactate enhancing the mitochondrial function and the role of lactate in HIIT-mediated mitochondrial quality control system in mouse hippocampus, the mitochondrial fusion and fission biomarker proteins expression were examined in primary cultured hippocampal cells following lactate treatment. Results showed that the expression of mitochondrial fusion protein MFN1 and MFN2 was significantly upregulated (*Z* = −1.96, *p* = 0.05; *Z* = −1.96, *p* = 0.05, Figures 9A,C,D), but the expression of OPA1 had no difference between the two groups (Figures 9A,B). Meanwhile, the expression of mitochondrial fission protein DRP1 and FIS1 was inhibited after lactate treatment (*Z* = −1.96, *p* = 0.05; *Z* = −1.96, *p* = 0.05, Figures 9E–G). These results verified that a high concentration of lactate could promote mitochondrial fusion and suppress mitochondrial fission.

### Lactate Weakly Influenced Mitophagy in Primary Hippocampal Cells

**FIGURE 10:**
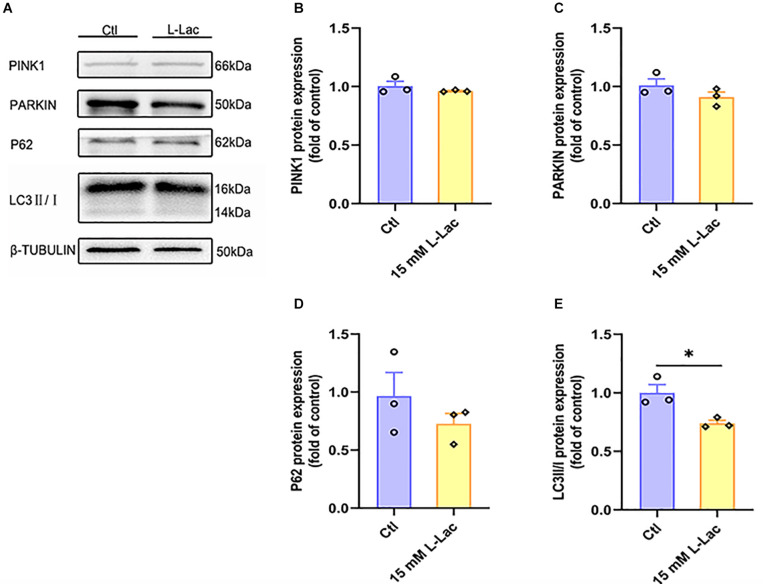
Lactate weakly influenced mitophagy in primary hippocampal cells. The PTEN-induced putative kinase 1 (PINK1), PARKIN, Sequestosome-1 (P62), and microtubule-associated protein light chain3 (LC3) were determined by WB to observe the effect of lactate on the mitophagy in primary hippocampal cells. **(A)** Representative WB image. **(B)** PINK1 protein expression. **(C)** PARKIN protein expression. **(D)** P62 protein expression. **(E)** LC3 protein expression. TUBULIN as the loading control. *N* = 3 in three independent experiments. ^∗^*P* ≤ 0.05 as compared with the Ctl group by non-parametric tests. Values are expressed as mean ± standard error of the mean.

After lactate treatment, the results showed that there was no difference between the two groups in the mitophagy related proteins expression of PINK1, PARKIN, and P62 (Figures 10A–D), except for the significantly downregulated protein expression of LC3II/I in the HIIT group (*Z* = −1.96, *p* = 0.05, Figures 10A,E). The results demonstrated that a high concentration of lactate had little effect on mitophagy, which were consistent with *in vivo* results.

### Lactate Enhanced Mitochondrial Biogenesis Signal and Increased Mitochondria DNA Copy Number in Primary Hippocampal Cells

**FIGURE 11:**
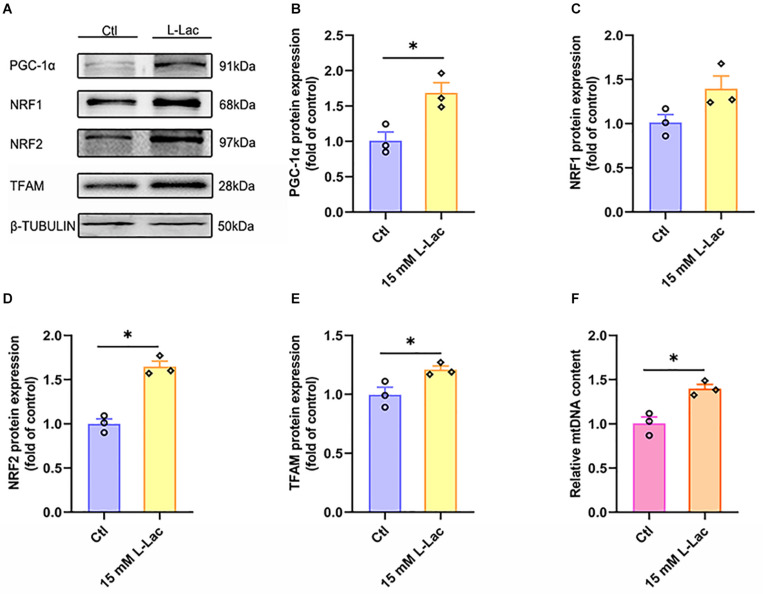
Lactate enhanced mitochondrial biogenesis signal and increased mitochondria DNA copy number in primary hippocampal cells. The peroxisome proliferator-activated receptor γ coactivator-1α (PGC-1α), nuclear respiratory factors 1 (NRF1), nuclear respiratory factors 1 (NRF2), and transcription factor A (TFAM) were determined by WB to observe the effect of lactate on mitochondrial biogenesis signals in primary hippocampal cells. The mt-Cytb level was determined by RT-PCR to evaluate mitochondria DNA copy number after the L-lactate treatment in primary hippocampal cells. **(A)** Representative WB image. **(B)** PGC-1α protein expression. **(C)** NRF1 protein expression. **(D)** NRF2 protein expression. **(E)** TFAM protein expression. **(F)** mt-Cytb level. TUBULIN and Cycs as the loading control. *N* = 3 in three independent experiments. ^∗^*P* ≤ 0.05 as compared with the Ctl group by non-parametric tests. Values are expressed as mean ± standard error of the mean.

PGC-1α, NRF1, NRF2, TFAM, and mitochondria DNA copy number were detected for evaluating the regulatory effect of lactate on mitochondrial biogenesis in primary cultured hippocampal cells. We found that both the protein expression of PGC-1α, NRF2, and TFAM (*Z* = −1.96, *p* = 0.05; *Z* = −1.96, *p* = 0.05; *Z* = −1.96, *p* = 0.05, Figures 11A,B,D,E) and the mitochondria DNA copy number (*Z* = −1.96, *p* = 0.05, Figure 11F) were increased after lactate treatment, except for the NRF1 protein expression (Figure 11C). The results verified that a high concentration of lactate could facilitate mitochondrial biogenesis and increase mitochondria DNA copy number, which was also consistent with *in vivo* results.

## Discussion

High-intensity interval training is a time-efficient alternative to moderate- or low-intensity continuous exercise for improving a range of physiological indexes associated with human health. HIIT has been widely used in public fitness, weight loss, and metabolic diseases treatment and rehabilitation (Kemi et al., 2005; Weston et al., 2014; Sperlich et al., 2017; Fealy et al., 2018). Beyond these, recent studies prove the protective effect of HIIT on brain function, especially in promoting BDNF expression (Saucedo Marquez et al., 2015; Tonoli et al., 2015; Boyne et al., 2019). Our results also confirmed that HIIT could promote hippocampal BDNF expression in healthy mice, which suggests HIIT has a protective effect on the brain function. Nevertheless, the mechanism of how HIIT influencing BDNF expression remains to be investigated.

High-intensity interval training is a typical anaerobic exercise, and glycolysis is the main energy supply in this process. Therefore, lactate level increases are the most obvious metabolic changes stimulated by HIIT and can be absorbed into neurons with the help of monocarboxylic acid transporters. MCT1 is primarily located in the endotheliocyte of the blood-brain barrier where it transports blood lactate into the brain (Gerhart et al., 1997; Pierre et al., 2000), and also so well as MCT4, located at the astrocytic membranes to transport intracellular lactate, produced by astrocytic glycolysis (Rafiki et al., 2003; Pierre and Pellerin, 2005; Bergersen, 2015). In an *in vivo* study, we found that HIIT promoted the mRNA and protein expression of MCT1 and MCT4, which may be the key reason for the increased hippocampal lactate levels. We also found that the level of blood lactate significantly increased to 6–10 mM in the HIIT group immediately after the training, while the blood lactate level was 2–4 mM in the Ctl group. Accordingly, hippocampal lactate increased (1.9 mM in the HIIT group vs. 1.4 mM in the Ctl group) immediately at the end of the exercise. Recently, the diverse roles of lactate in mediating brain function have been well characterized (Berthet et al., 2009; Lauritzen et al., 2014; Tang et al., 2014; Mosienko et al., 2015; Dienel, 2017; Magistretti and Allaman, 2018), such as mediating synaptic plasticity (Schurr et al., 1988; Yang et al., 2014; Margineanu et al., 2018; El Hayek et al., 2019; Scavuzzo et al., 2020), cerebral microvasculogenesis (Morland et al., 2017; Boitsova et al., 2018), and neuronal activity (Bozzo et al., 2013; Tang et al., 2014; Herrera-Lopez and Galvan, 2018; de Castro Abrantes et al., 2019). Therefore, we speculated that the lactate might be an intermediary between HIIT and increased hippocampal BDNF expression. Although a low sample size limits the evaluation of the positive effect of lactate on BDNF, our results show that 15 mM lactate can induce the BDNF protein expression, which is in line with the previous studies (Margineanu et al., 2018; El Hayek et al., 2019). Therefore, we think that lactate may be one of the most important factors contributing to the positive effect of HIIT on the BDNF expression in the mouse hippocampus.

Of note, BDNF biosynthesis requires a great deal of ATP (Gomez-Pinilla, 2008; Vignoli and Canossa, 2017; Hu et al., 2020), and 95% of ATP is derived from mitochondrial oxidative phosphorylation (OXPHOS) (Nakamura and Lipton, 2017; Ould Amer and Hebert-Chatelain, 2018). Four large protein complexes (I, II, III, and IV) and ATP synthase in the inner mitochondrial membrane are the pivotal links that affect the efficiency of ATP production by OXPHOS. The three proton-pumping complexes of the electron transfer chain are complexes I, III, and IV. Complex II does not pump protons but contributes reduced ubiquinone. The flow of protons back into the matrix via a proton channel in the ATP synthase leads to conformational changes in the nucleotide-binding pockets and the formation of ATP (Kühlbrandt, 2015). Previous studies found that exercise, even moderate-intensity exercise (Bayod et al., 2011; Masaki Takimoto, 2014) or high-intensity exercise (Lezi et al., 2014), can increase brain mitochondrial OXPHOS. Our research results showed that HIIT promoted hippocampal COX5b gene (complex IV) expression and that a high concentration of lactate enhanced cultured hippocampal cell Uqcrc1 (complex III) and Atp5a1 (ATP synthase) gene expression. In addition, both HIIT and high concentrations of lactate significantly elevated ATP levels. These results suggested that HIIT probably acts through lactate to increase hippocampal mitochondrial OXPHOS and energy generation efficiency to meet high brain energy demands, such as BDNF production. Then, the expression of BDNF may further contribute to the beneficial effects of exercise on synaptic plasticity (Raefsky and Mattson, 2017). However, the question of how lactate promotes mitochondrial function to induce BDNF expression needs to be solved.

As described in previous studies, 2 weeks of intraperitoneal administration of 18 mM lactate can mimic the effect of 7 weeks of exercise on improving PRC expression and increasing mtDNA copy number in the mice brain (Lezi et al., 2013). Furthermore, 20 mM lactate was proven to stimulate the protein expression of PGC-1α in mouse neurons (El Hayek et al., 2019) and regulate mitochondrial biosynthesis signals in L6 cells (Hashimoto et al., 2007). Hence, lactate is the pivotal element for mitochondrial biogenesis and thus mitochondrial function improvement. Moreover, physical exercise has been confirmed to increase the expression of proteins involved in mitochondrial fusion and biogenesis, decrease the expression of the mitochondrial fission-related protein DRP1, and simultaneously alter mitophagy markers. All of the changes in the mitochondrial quality control system in the brain are beneficial for increasing mitochondrial function and enhancing brain function (Marques-Aleixo et al., 2015; Luo et al., 2017). Considering the effect that the influence of exercise on mitochondrial function is guaranteed by the mitochondrial quality control system, we speculate that lactate may also participate in mediating this system to maintain the mitochondrial function during HIIT. The mitochondrial quality control system regulates bioenergetic efficiency and energy expenditure. The system includes mitochondrial fusion, fission, mitophagy, and mitochondrial biogenesis. Normally, mitochondria are dynamic organelles that continually fuse and divide, which determines mitochondrial morphology and allows their immediate adaptation to energetic needs. Mitochondrial fusion can repair dysfunctional mitochondria by mixing the contents with healthy mitochondria to regulate MFN1, MFN2, and OPA1 (Rovira-Llopis et al., 2017). Studies show that acute or chronic exercise increases energy demand and significantly promotes MFN2 and OPA1 protein expression. Then, the mitochondria show obvious fusion tendencies. With the enhancement of fusion, the levels of OXPHOS and ATP complexes are significantly elevated, resulting in an increase in ATP levels (Yao et al., 2019). Mitochondrial fission segregates badly damaged mitochondria, which is regulated by DRP1 and mitochondrial FIS1 (Cai and Tammineni, 2016). Under the stimulation of various factors, DRP1 is recruited into the mitochondria and undergoes oligomerization. Several DRP1 molecules closely surround the mitochondria to form a ring structure, which hydrolyzes GTP depending on the activity of its GTPase, causing the inner and outer membranes of mitochondria to break and leading to mitochondrial fission (Ji et al., 2015). Reduced fusion and increased mitochondrial fission may be the primary causes of mitochondrial dysfunction and neuronal damage. In this study, we found that both HIIT and high concentrations of lactate upregulated the protein expression of OPA1, MFN1, and MFN2 to promote mitochondrial fusion but significantly downregulated the protein expression of DRP1 and FIS1 to inhibit mitochondrial fission. The results demonstrated that there was a very high energy requirement in the hippocampus in HIIT, and the significant increase in OXPHOS levels was also the result of enhanced mitochondrial fusion. More importantly, the results verified that lactate may participate in regulating HIIT-triggered hippocampal mitochondrial fusion and fission.

In addition, mitophagy plays a critical role in maintaining mitochondrial quality by degrading aging, damaged, or dysfunctional mitochondria (Tang et al., 2016). Damage to the system that regulates mitophagy can lead to the accumulation of dysfunctional mitochondria, which is a characteristic of aging-related diseases such as Alzheimer’s disease and Parkinson’s disease. Among the multiple mechanisms by which mitochondria are targeted for degradation at the autophagosome, the best understood and the classical pathway is PINK1-PARKIN-dependent mitophagy. In the presence of damaged mitochondria, PINK1 accumulates on the outer mitochondrial membrane and selectively recruits cytosolic PARKIN, known as a cytosolic E3-ubiquitin ligase, to mitochondria (Chen and Dorn, 2013). Then, PARKIN ubiquitinates the depolarized mitochondria and binds to LC3 to promote the assembly of the autophagic machinery and eliminate damaged mitochondria (Lee et al., 2010). Furthermore, P62 is recruited to mitochondria in a PARKIN- and depolarization-dependent manner. Knockdown of P62 substantially inhibits mitophagy. However, one study noted that P62 recruitment to mitochondria may also be insufficient for mitophagy (Narendra et al., 2010). Anyway, PINK1 and PARKIN, act as the mitochondrial gatekeepers, can sense healthy versus unhealthy mitochondria and can regulate mitochondrial quality control pathways (Leites and Morais, 2018). However, the effect of HIIT or lactate on hippocampal mitophagy remains unknown. Our results showed that both HIIT and high concentrations of lactate had almost no effect on hippocampal mitophagy; namely, HIIT did not damage hippocampal mitochondria, which also suggested that HIIT is safer for improving cognitive function and brain health.

Mitochondrial biosynthesis is a dynamic process in which new mitochondria form to maintain and restore mitochondrial structure, quantity and function under the conditions of increased energy demand. PGC-1α is considered as the master regulator of mitochondrial biogenesis because it interacts with two key nuclear transcription factors, NRF1 and NRF2, and it increases the expression levels and activities of NRF1 and NRF2 through protein-protein interactions. NRF1 and NRF2 activate mitochondrial TFAM and bind to promoter regions of nuclear genes encoding subunits of the five complexes in the mitochondrial electron transport chain, thereby increasing mtDNA replication (namely, mtDNA copy number) for mitochondrial biogenesis (Sharma et al., 2013). The mitochondrion is the only organelle that contains its own DNA (mtDNA) outside the nucleus, and mtDNA copy number reflects the abundance of mitochondria within a cell and the cellular energy status of tissues. Generally, in mitochondrial diseases, a mutation in the mtDNA leads to a loss of functionality of the OXPHOS, and that can lead to a depletion of ATP, which can, in turn, induce further mtDNA mutations. One study showed that high-intensity exercise, a kind of supra-lactate threshold exercise, increased hippocampal mtDNA copy number and activated a partial mitochondrial biogenesis in aged mice, which may benefit protection against decreased cognitive function in the aging brain (Lezi et al., 2014). Here, we also found that both HIIT and high concentrations of lactate treatment could promote hippocampal PGC-1α and NRF2 protein expression and increase mtDNA copy number, which demonstrated that HIIT could facilitate hippocampal mitochondrial biogenesis through lactate to accommodate high energy requirements. Additionally, we found an interesting phenomenon in which the protein expression of NRF1 only had an increasing tendency but no significant difference following HIIT or lactate treatment. These *in vitro* results were consistent with previous research conclusions (Hashimoto et al., 2007). As described above, our results elucidated that lactate participated in mediating the effect of HIIT on mitochondrial biogenesis in the mouse hippocampus by activating PGC-1α- NRF2 signals.

In this study, we found 6-week HIIT through elevating hippocampal lactate levels could regulate hippocampal mitochondrial quality control system to improve mitochondrial function, which may then promote BDNF expression. Notably, the potential mechanism is unknown how the increased hippocampal lactate regulates mitochondrial fusion, fission, and biosynthesis during HIIT. Nonetheless, our results suggest that HIIT is beneficial for hippocampal mitochondrial function and BDNF expression, and lactate plays the vital signaling role. However, it should be noted that the mice hippocampal cell culture experiment with 15 mM lactate treatment for 3 h is not enough to represent what has happened actually in peripheral and central physiological changes of lactate induced by HIIT. Different mode of HIIT and the characteristics of rapid metabolism of lactate *in vivo* will inevitably make the blood and brain lactate level flexible. Therefore, it is worth considering whether lactate itself could serve as the effective exercise mimetic to exert the effect of enhancing brain mitochondrial function. Although lactate is not completely responsible for all exercise effect, it does regulate the mitochondrial quality control system in hippocampal cells. It is of significance to investigate the link of lactate, exercise, and brain mitochondrial function because that may help us understand how exercise benefits brain health.

## Conclusion

This is the first study to demonstrate that 6-week HIIT can enhance mice hippocampal mitochondrial fusion and biogenesis and inhibit mitochondrial fission to lift mitochondrial energy production function, which may promote the BDNF expression. This process is mainly attributed to the novel signal role of lactate. However, there are some limits in this study. Firstly, the energy substrate role of lactate in mitochondrial function cannot be excluded because lactate converts into pyruvate for mitochondrial OXPHOS and ATP production. In the future, we will focus on how this dual role of lactate influences mitochondrial function in the brain. Secondly, our results cannot show an obvious evidence that HIIT-mediated mitochondrial quality control system changes would directly contribute to the expression of BDNF.

## Data Availability Statement

The raw data supporting the conclusions of this article will be made available by the authors, without undue reservation.

## Ethics Statement

The animal study was reviewed and approved by the Ethics Committee for Science Research at Shanghai.

## Author Contributions

JH completed experiment and drafted the manuscript. MC assisted with drafting manuscript and drawing the graph. QS, ZL, YF, BL, and XX assisted with keeping animals and partial experiment. SL conceptualized the article and revised the final version. All authors read and approved the final manuscript.

## Conflict of Interest

The authors declare that the research was conducted in the absence of any commercial or financial relationships that could be construed as a potential conflict of interest.
