# Supplementary material for: Elevated Lactate by High-Intensity Interval Training Regulates the Hippocampal BDNF Expression and the Mitochondrial Quality Control System
Source: Front Physiol. 2021 Feb 25;12:629914. doi: 10.3389/fphys.2021.629914 (PMC7946986; doi:10.3389/fphys.2021.629914)
Supplement: Supplementary file 1 [file Data_Sheet_1.PDF]

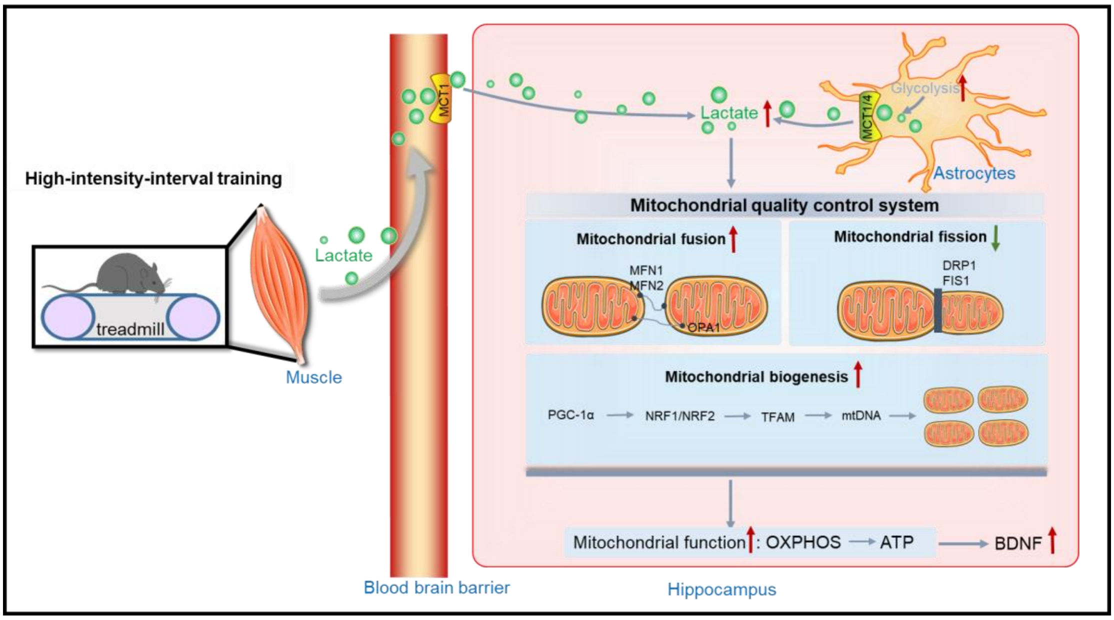

Figure 12. Graphical summary of lactate action as the exercise mimetic to improve BDNF expression via regulating the mitochondrial quality control system. During HIIT, the high level of blood lactate, produced by muscle contraction, will cross into brain by MCT1. Meanwhile, lactate is also produced by the glycolysis in astrocytes and transported into extracellular space by MCT4 and MCT1 in mice hippocampus. Lactate can work as the signal molecule to regulate hippocampal mitochondrial quality control system, including promoting mitochondrial fusion, inhibiting mitochondrial fission, and activating mitochondrial biogenesis related signaling pathway. As the result, the mitochondrial function was significantly improved and thus BDNF expression level was markedly enhanced in mice hippocampus.
