# Supplementary figures and images for: Elevated Lactate by High-Intensity Interval Training Regulates the Hippocampal BDNF Expression and the Mitochondrial Quality Control System
Source: Front Physiol. 2021 Feb 25;12:629914. doi: 10.3389/fphys.2021.629914 (PMC7946986; doi:10.3389/fphys.2021.629914)

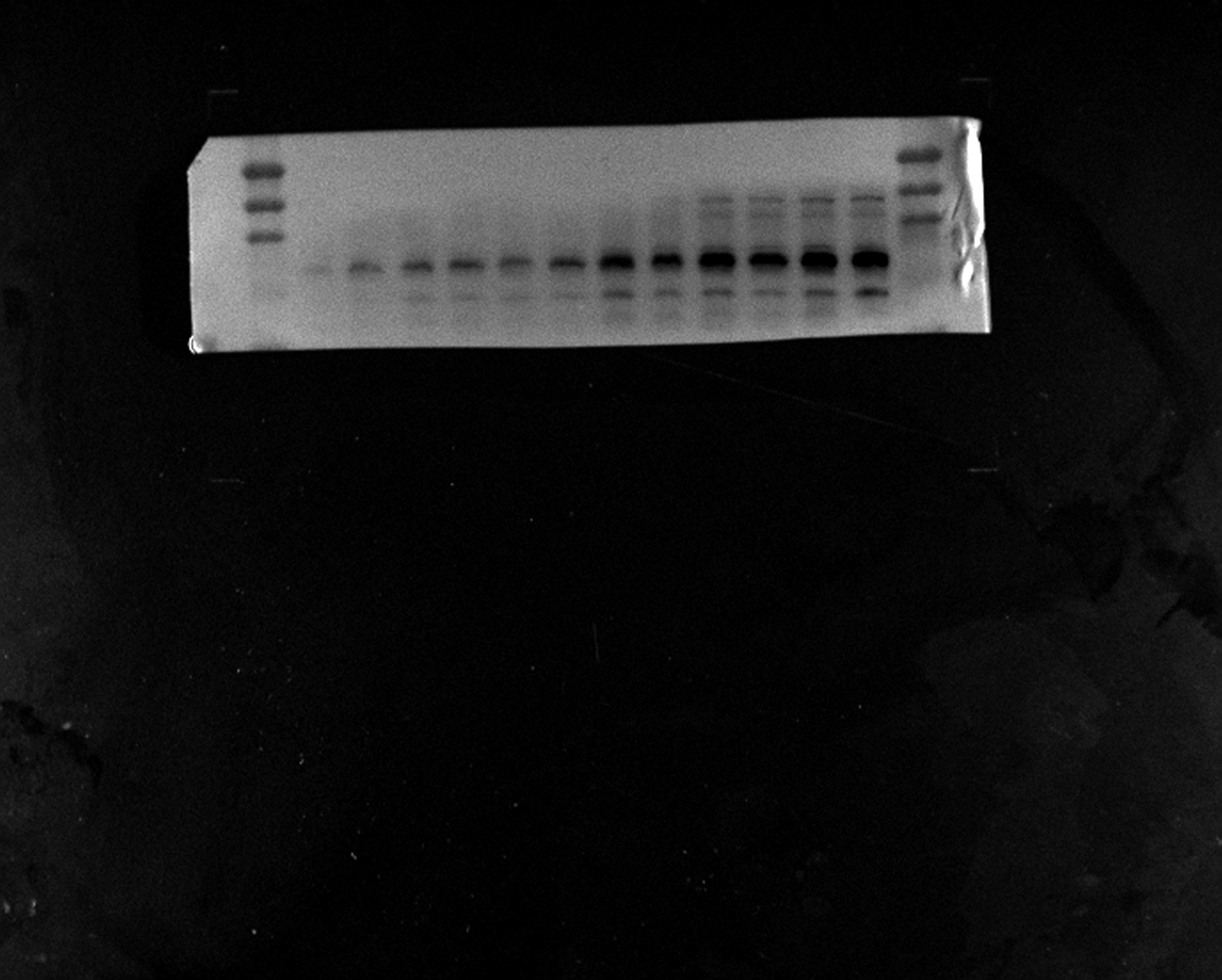

Supplement: Supplementary file 2 [file Data_Sheet_2.ZIP › animals WB/BDNF 3S SHANGMIANDAI(70,50,40,35,25,20)-HCC.tif]

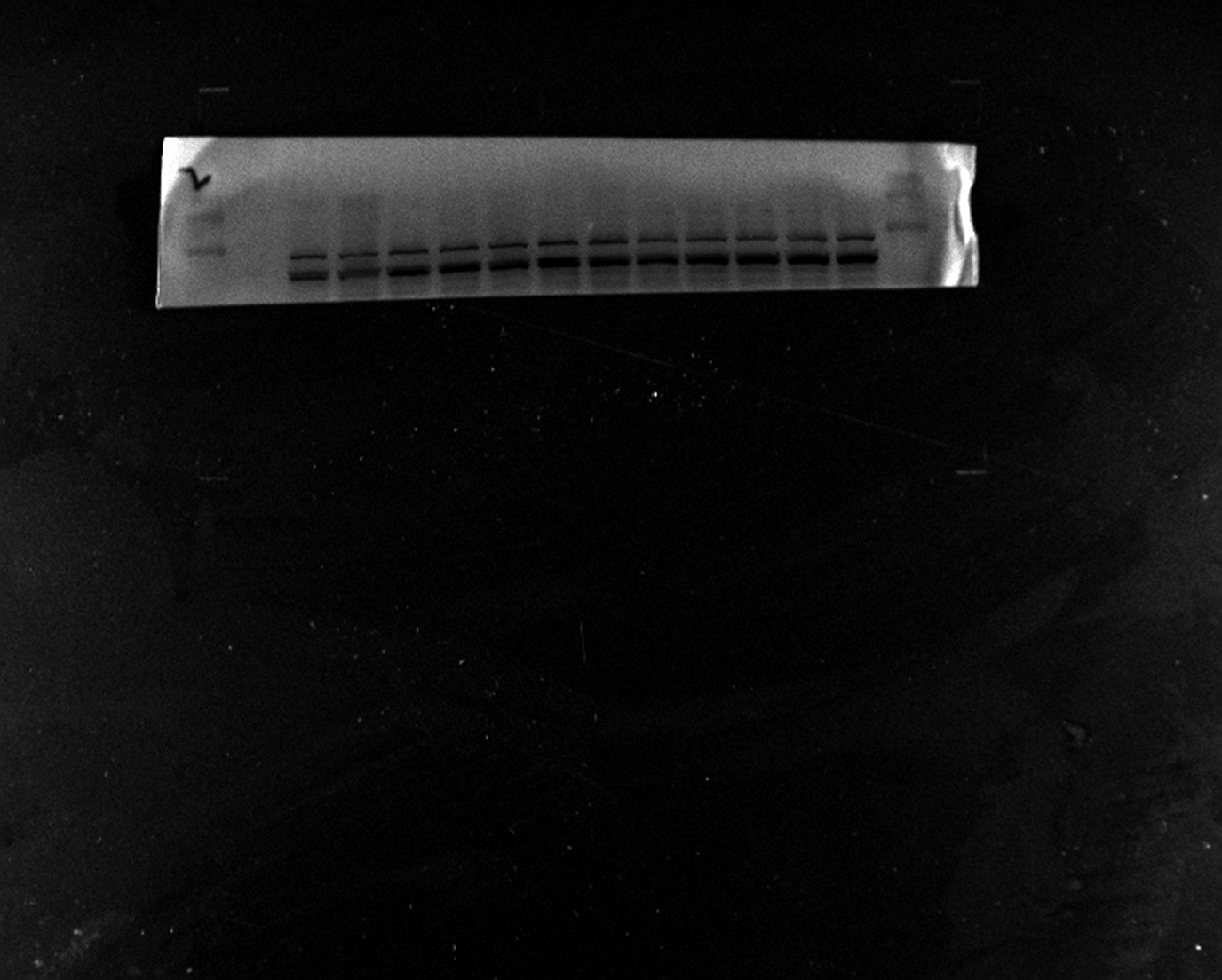

Supplement: Supplementary file 2 [file Data_Sheet_2.ZIP › animals WB/DRP1 30S XIAMIAM(70CAI)-HC.tif]

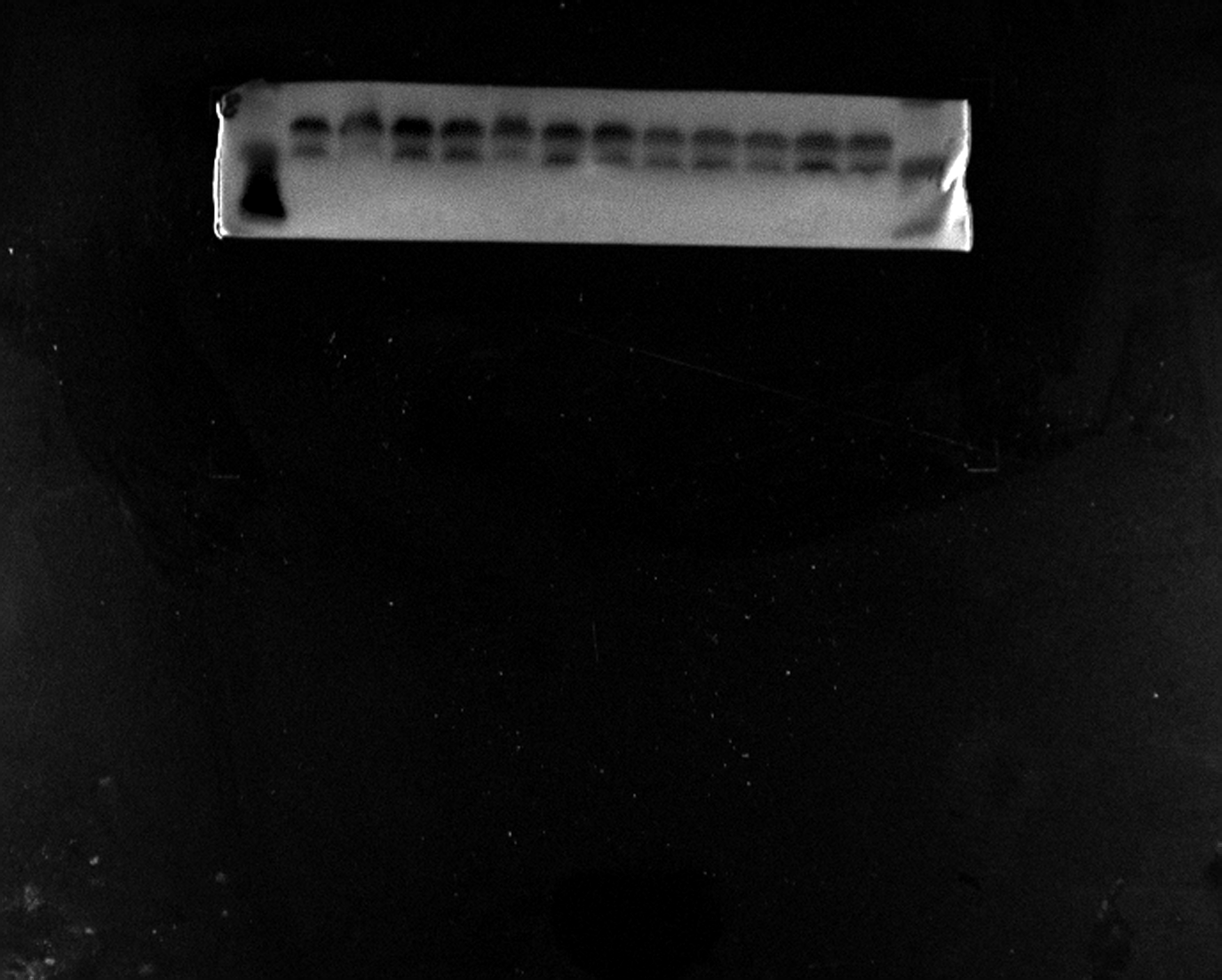

Supplement: Supplementary file 2 [file Data_Sheet_2.ZIP › animals WB/FIS 1S ZUISHANGMIAN-HC.tif]

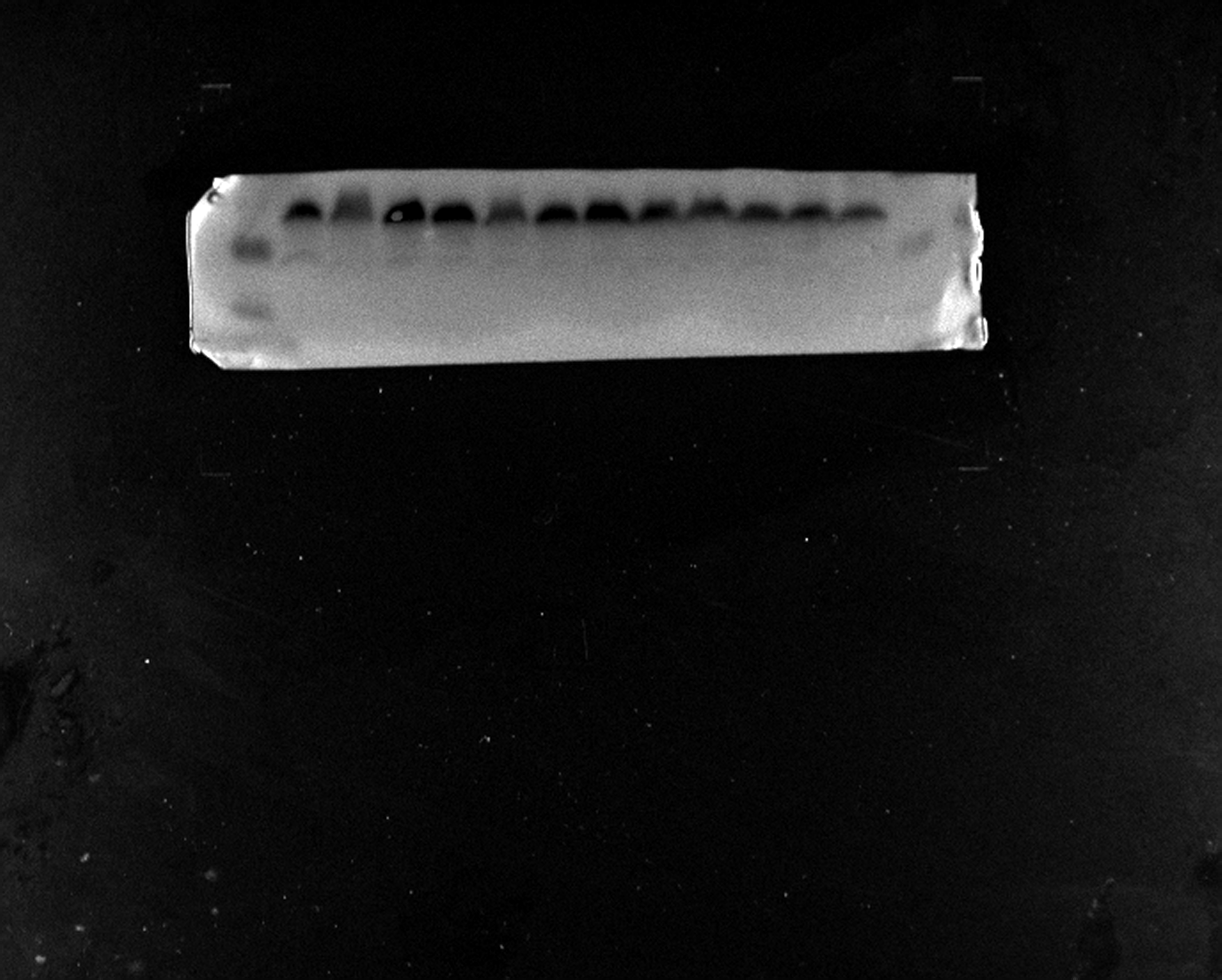

Supplement: Supplementary file 2 [file Data_Sheet_2.ZIP › animals WB/LC3 3S-HC.tif]

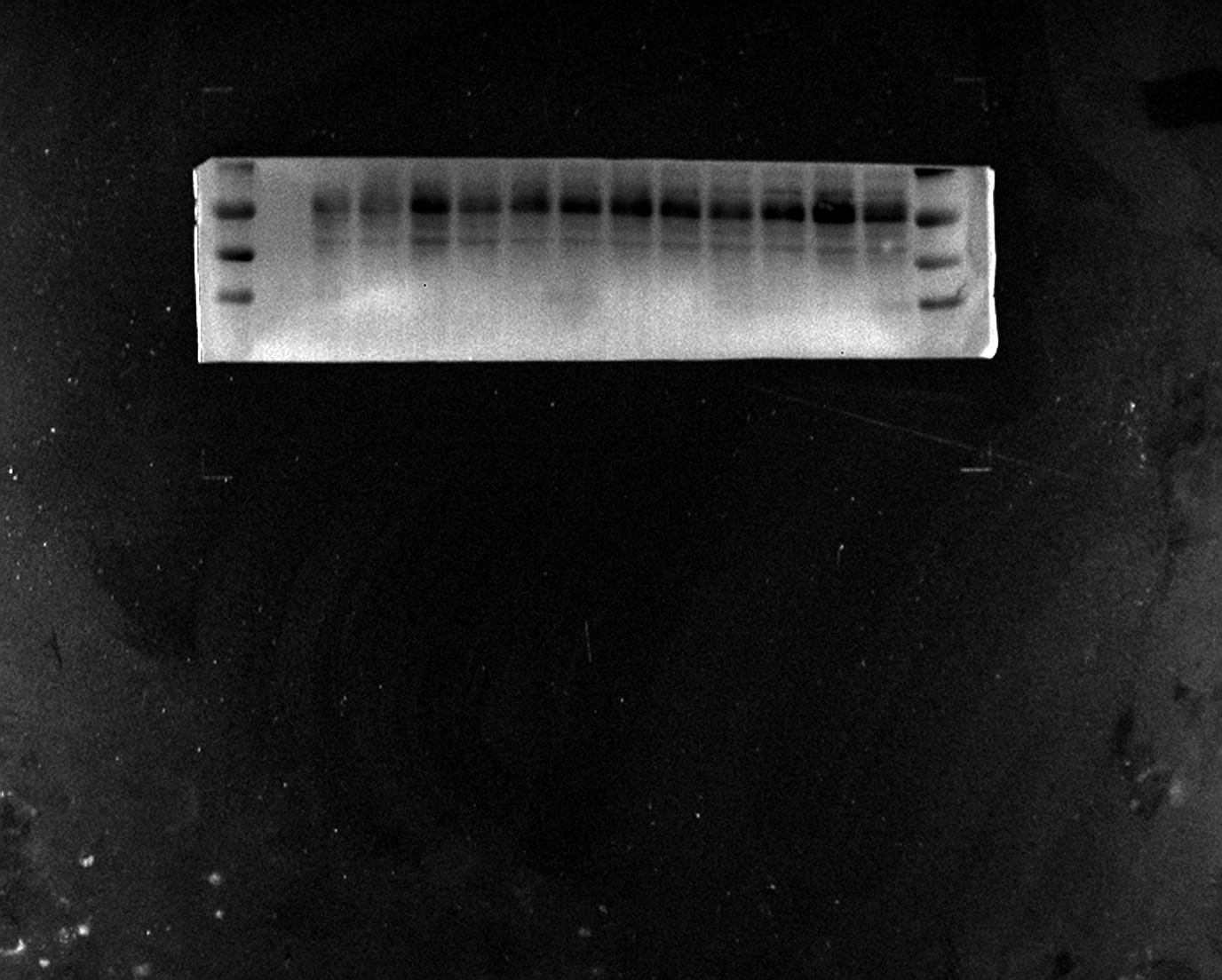

Supplement: Supplementary file 2 [file Data_Sheet_2.ZIP › animals WB/MCT1.tif]

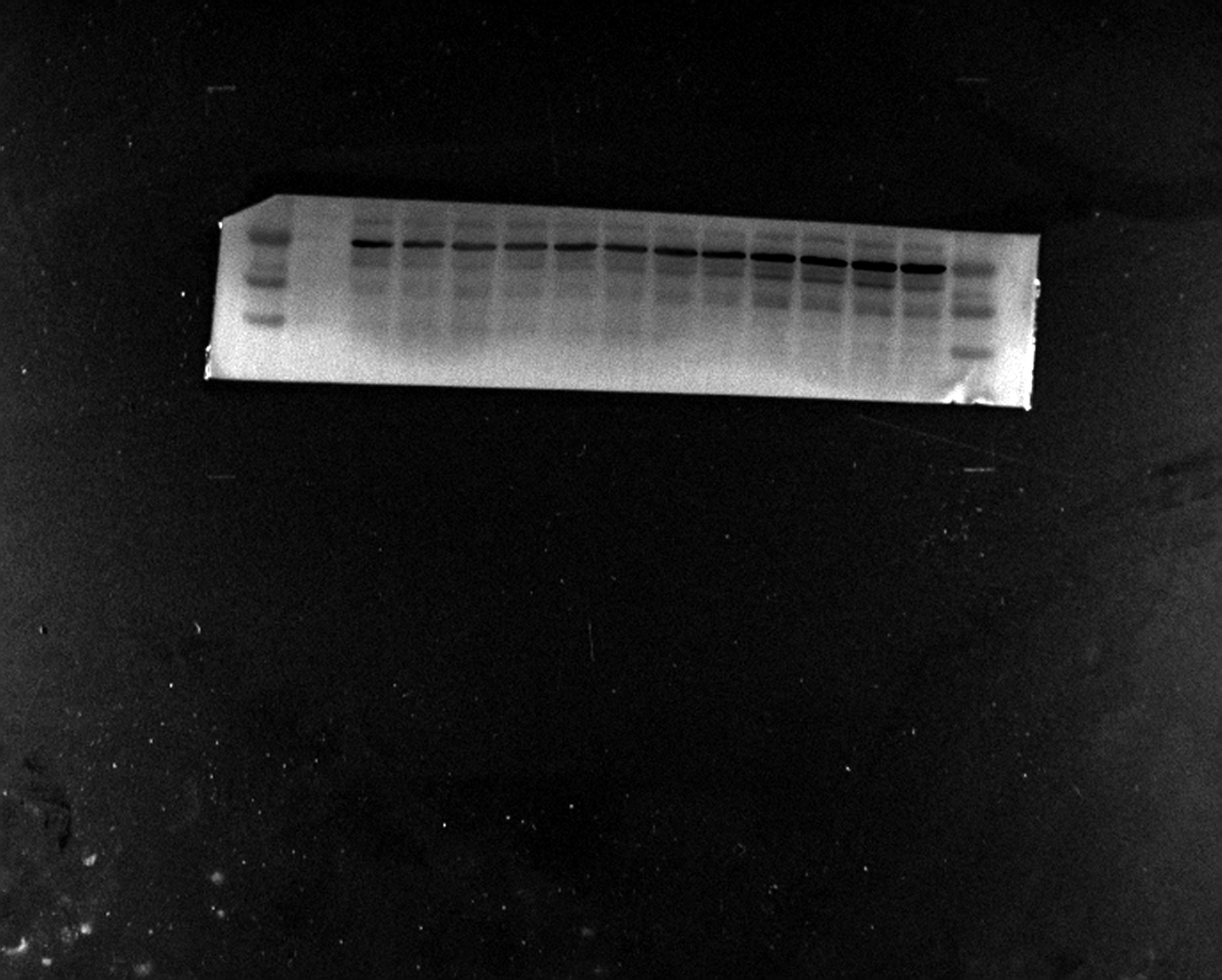

Supplement: Supplementary file 2 [file Data_Sheet_2.ZIP › animals WB/MCT4.tif]

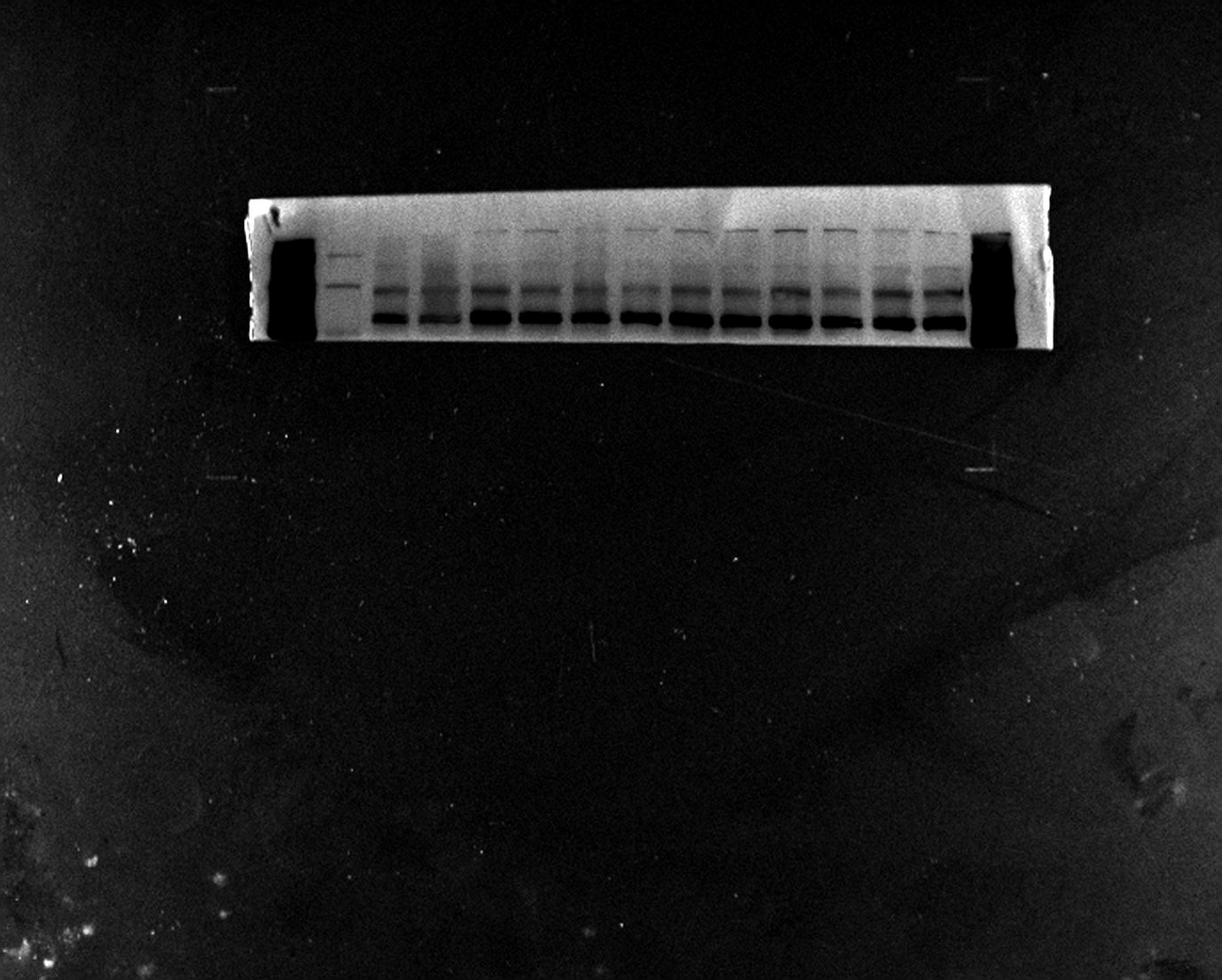

Supplement: Supplementary file 2 [file Data_Sheet_2.ZIP › animals WB/MFN1 60S-HC(70CAI, XIAMIANTIAODAI).tif]

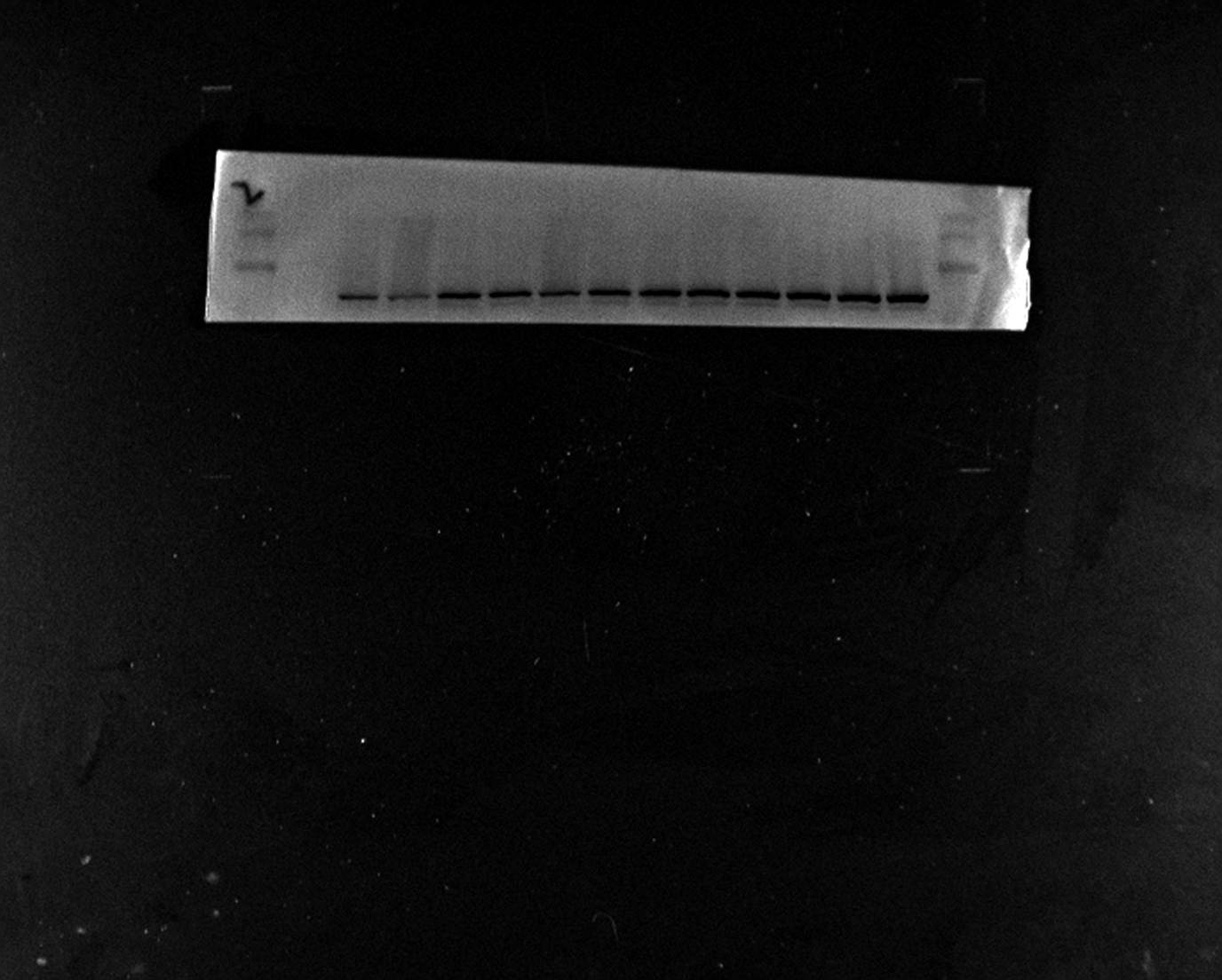

Supplement: Supplementary file 2 [file Data_Sheet_2.ZIP › animals WB/MFN2 animals.tif]

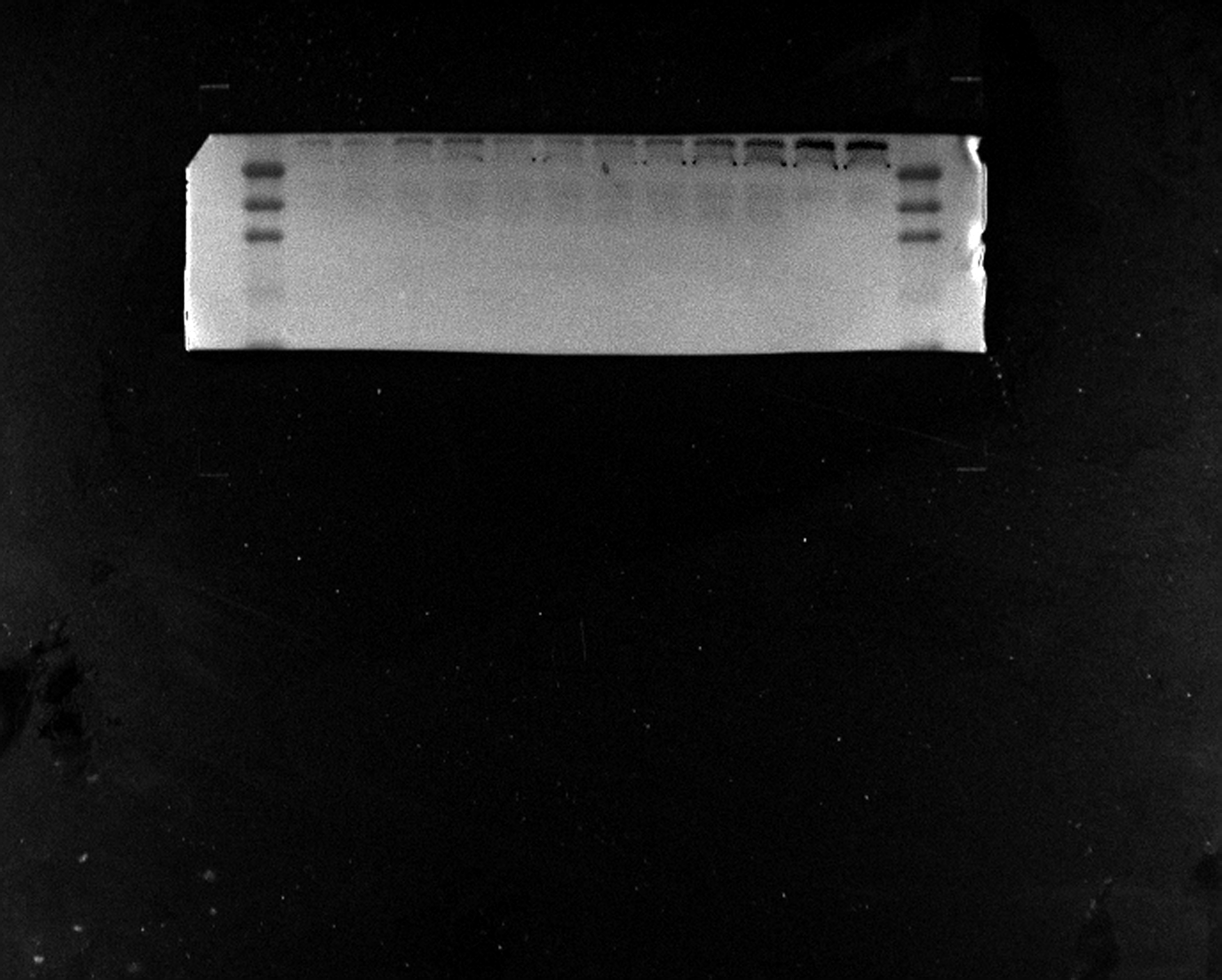

Supplement: Supplementary file 2 [file Data_Sheet_2.ZIP › animals WB/NRF1animal.tif]

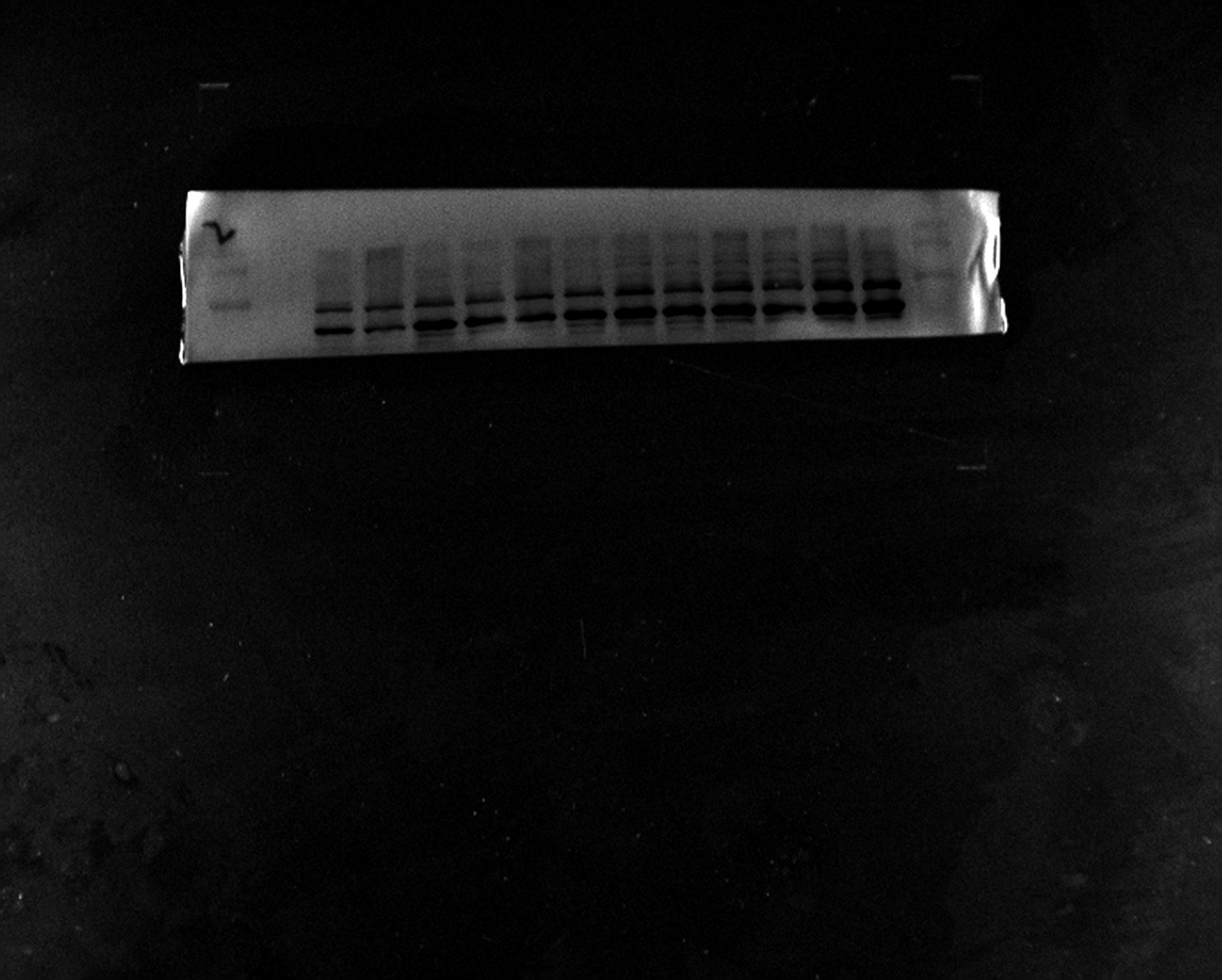

Supplement: Supplementary file 2 [file Data_Sheet_2.ZIP › animals WB/NRF2 3MIN(SHANGMIANDAI)-HC.tif]

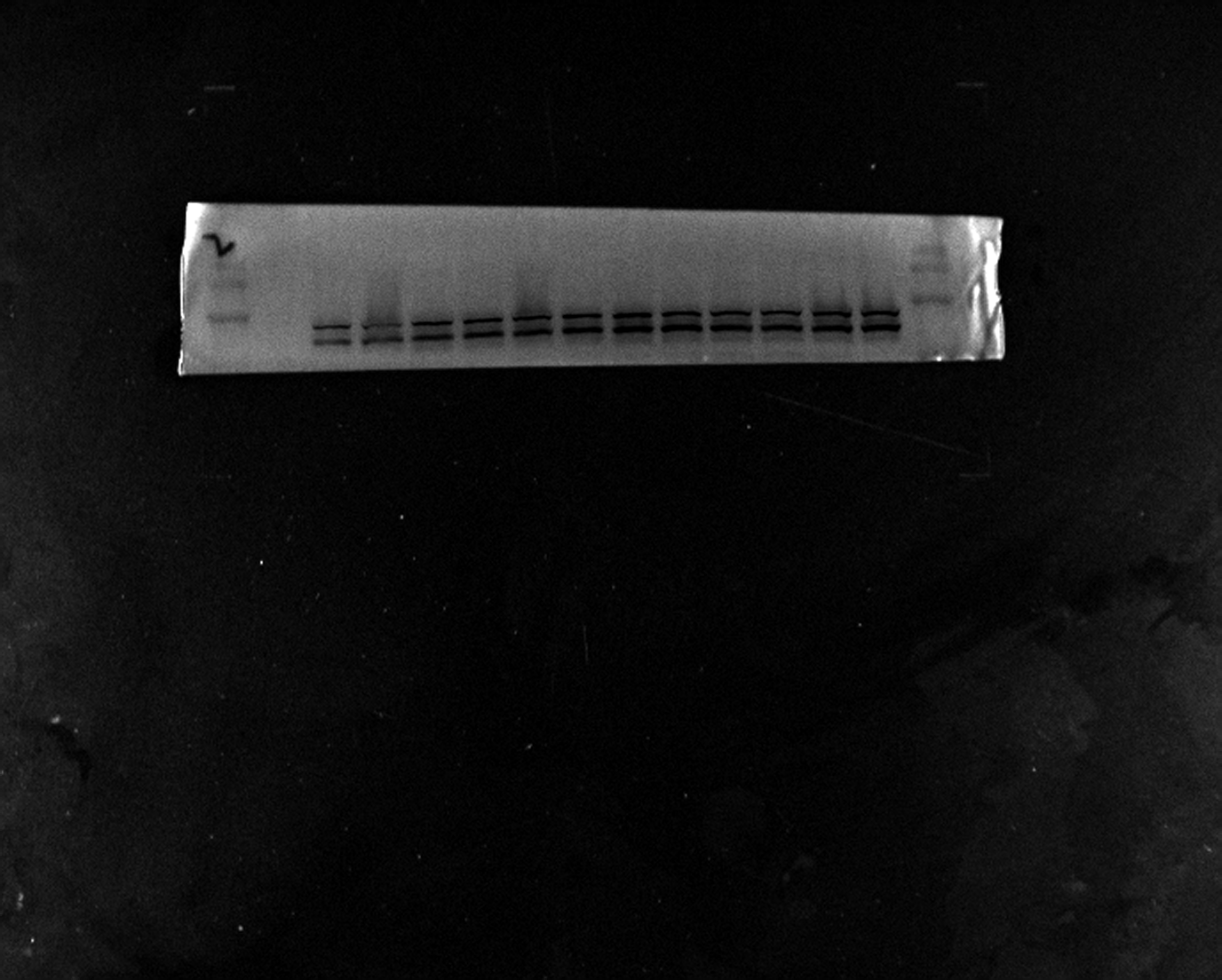

Supplement: Supplementary file 2 [file Data_Sheet_2.ZIP › animals WB/OPA1 animals.tif]

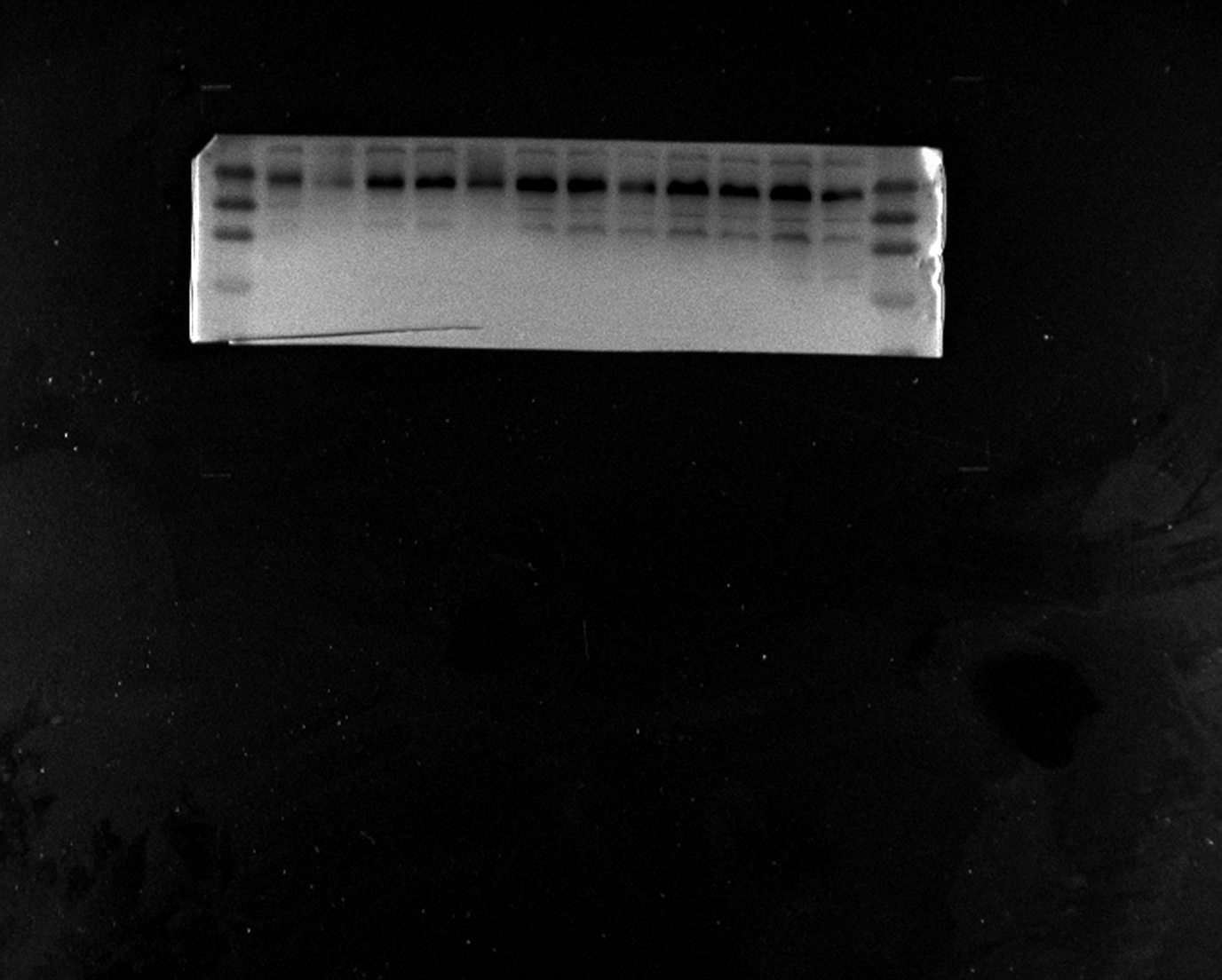

Supplement: Supplementary file 2 [file Data_Sheet_2.ZIP › animals WB/PARKIN 5MIN(DI2TIAO, 70-20,MARKER 50)-HC.tif]

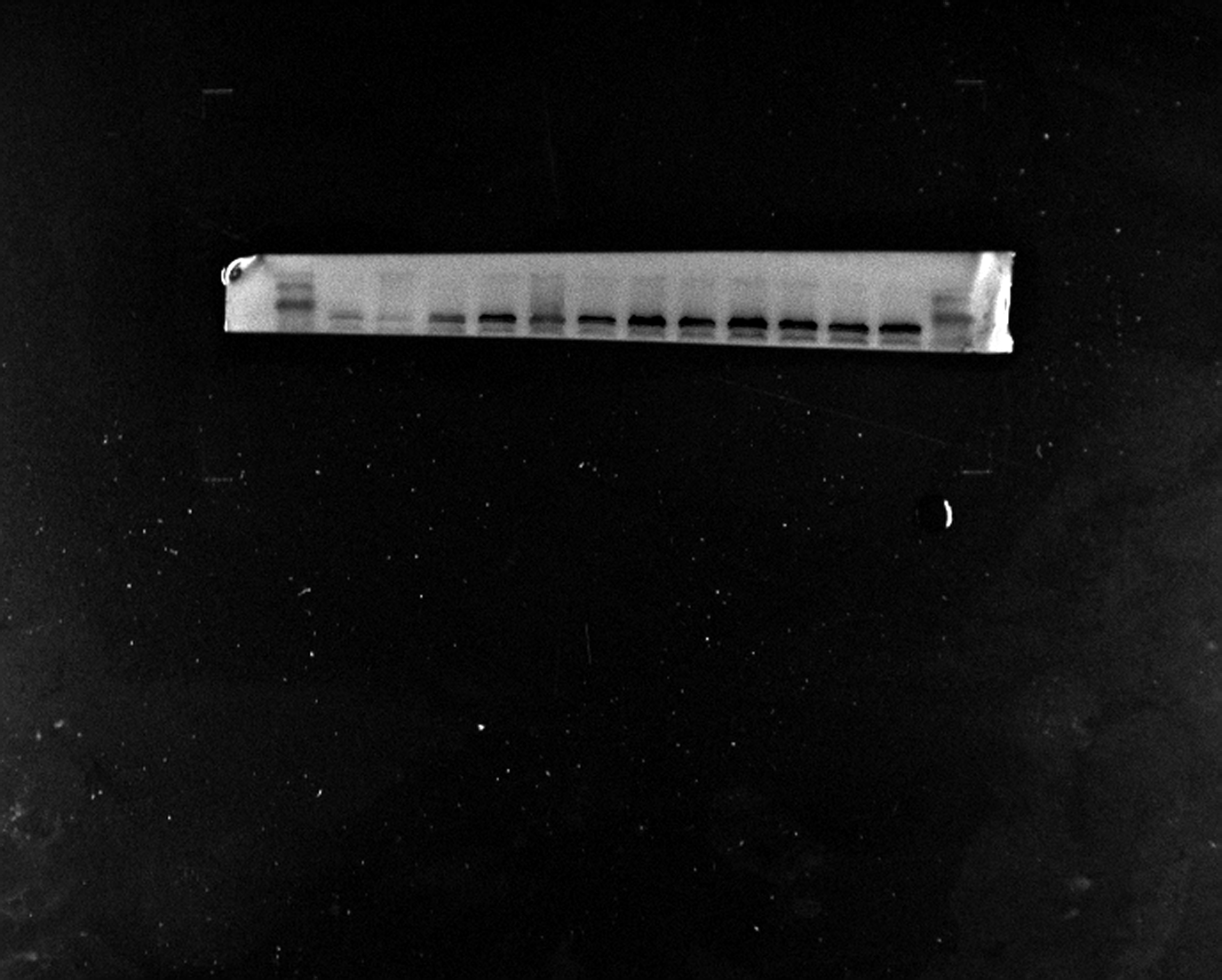

Supplement: Supplementary file 2 [file Data_Sheet_2.ZIP › animals WB/PGC-1animal.tif]

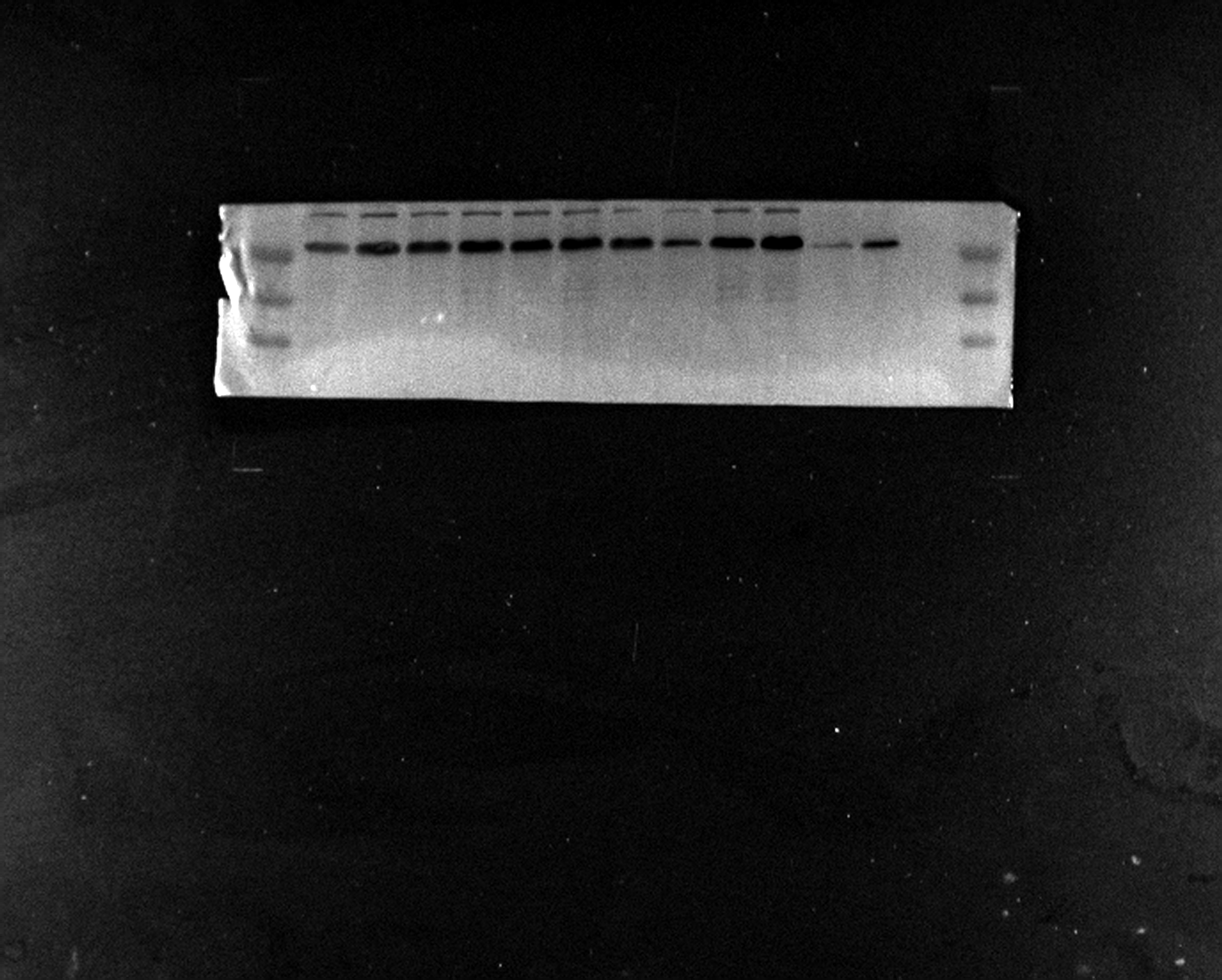

Supplement: Supplementary file 2 [file Data_Sheet_2.ZIP › animals WB/PINK 1animal.tif]

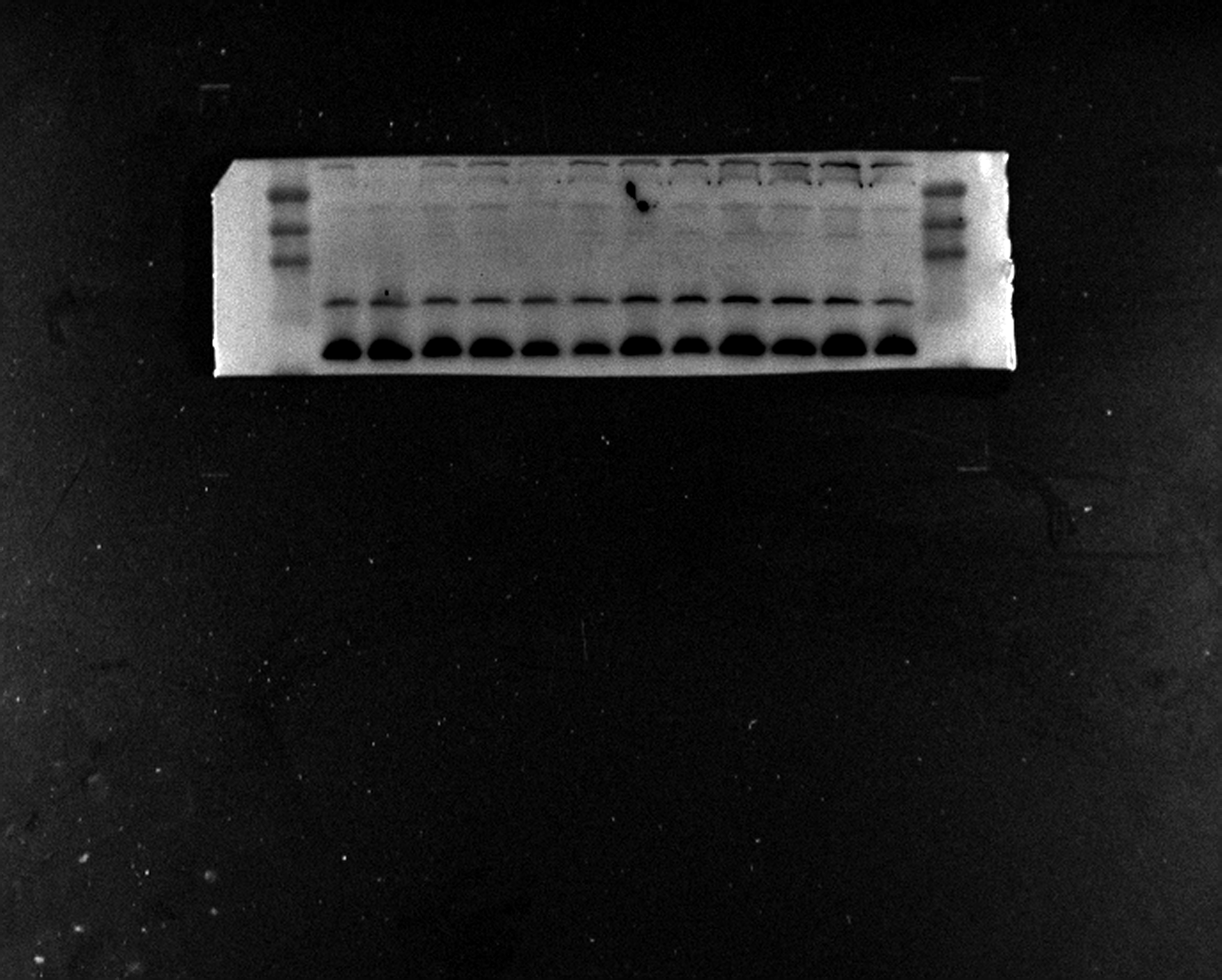

Supplement: Supplementary file 2 [file Data_Sheet_2.ZIP › animals WB/TFAM 30S(70-10,SHANGMIANTIAODAI )-HC.tif]

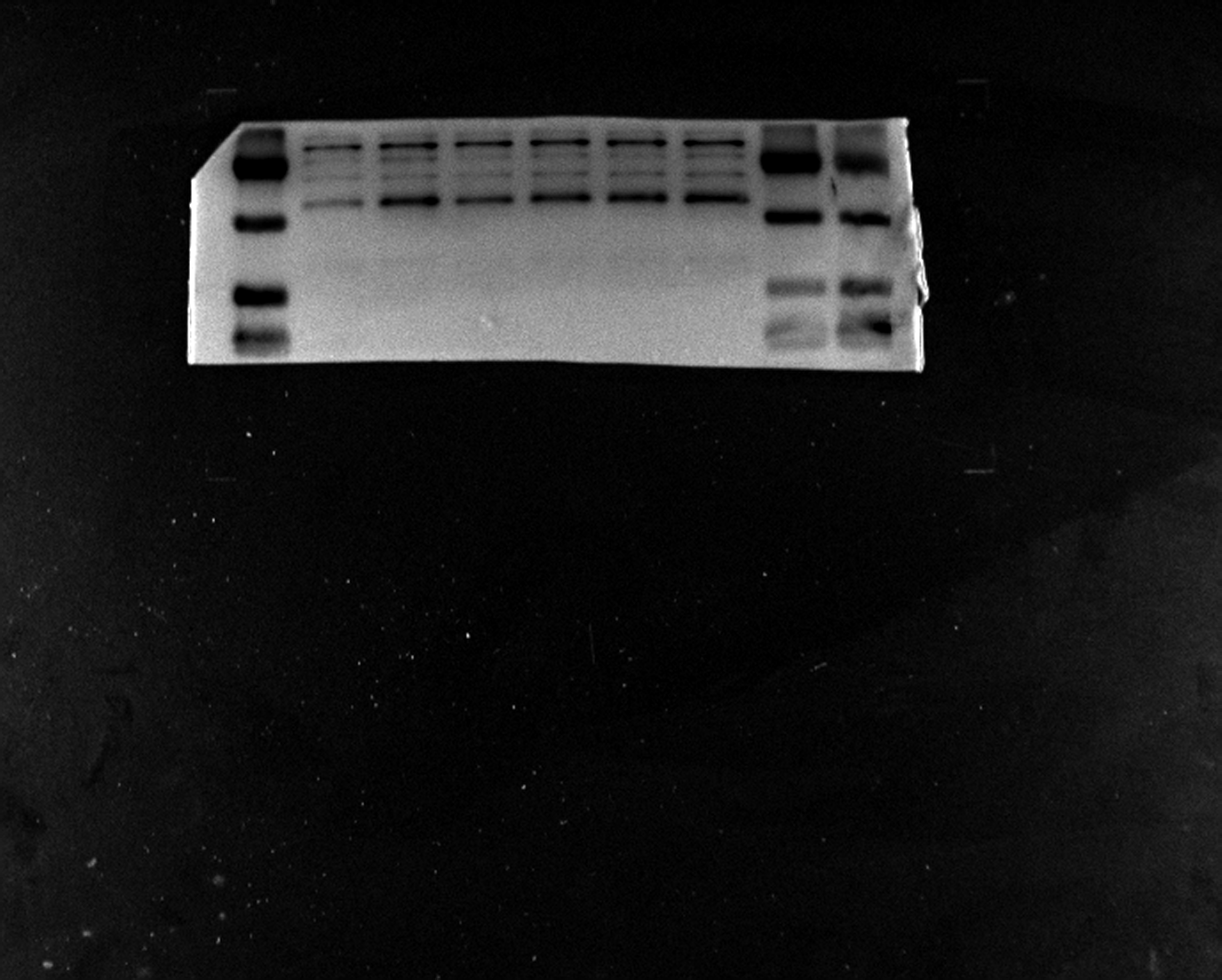

Supplement: Supplementary file 3 [file Data_Sheet_3.ZIP › cells for WB/BDNF 60S(35-10,ZUISHANGMIANTIAODAI)-HC.tif]

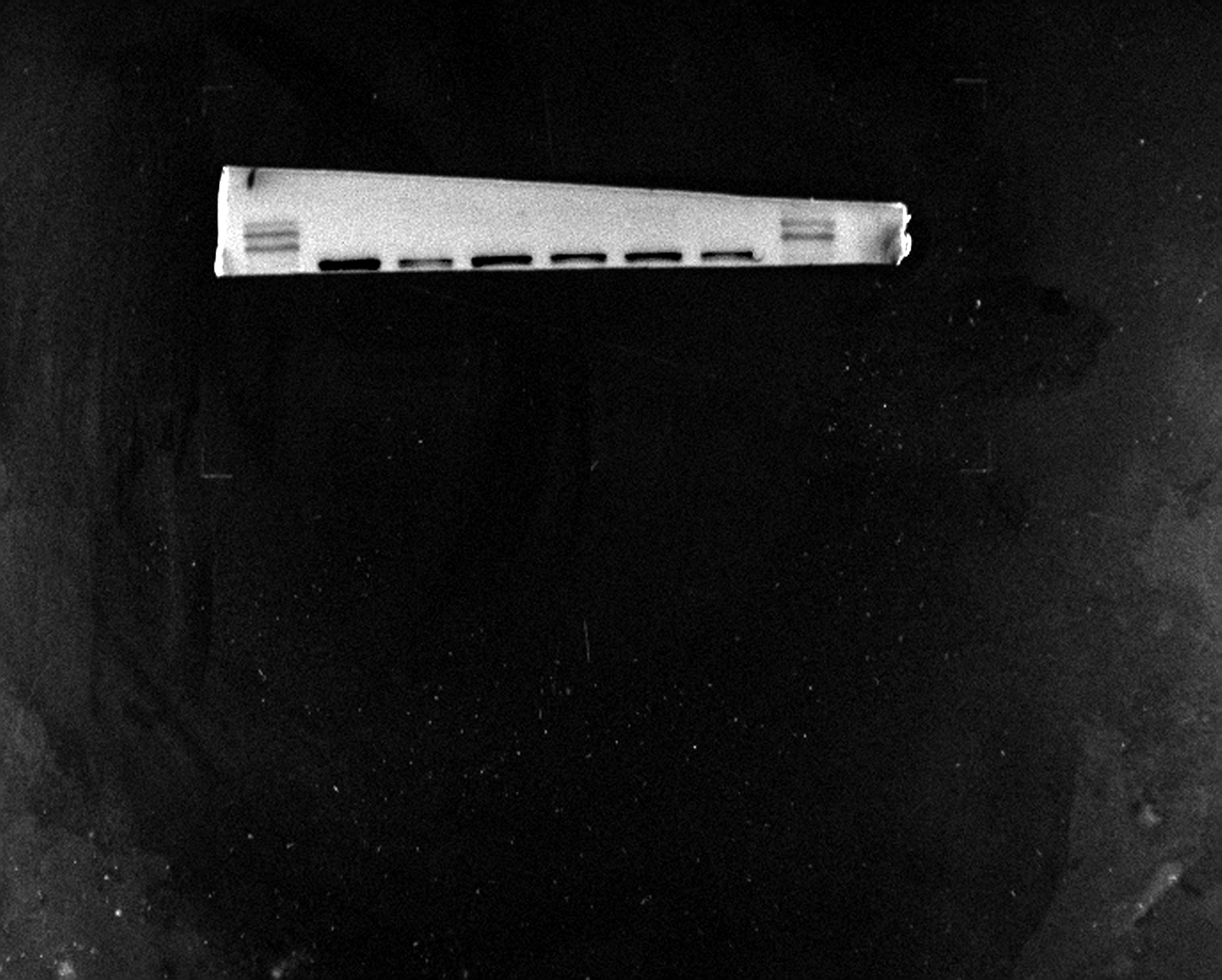

Supplement: Supplementary file 3 [file Data_Sheet_3.ZIP › cells for WB/DRP1(70CAI,250-150-100-70) 3MIN-HC.tif]

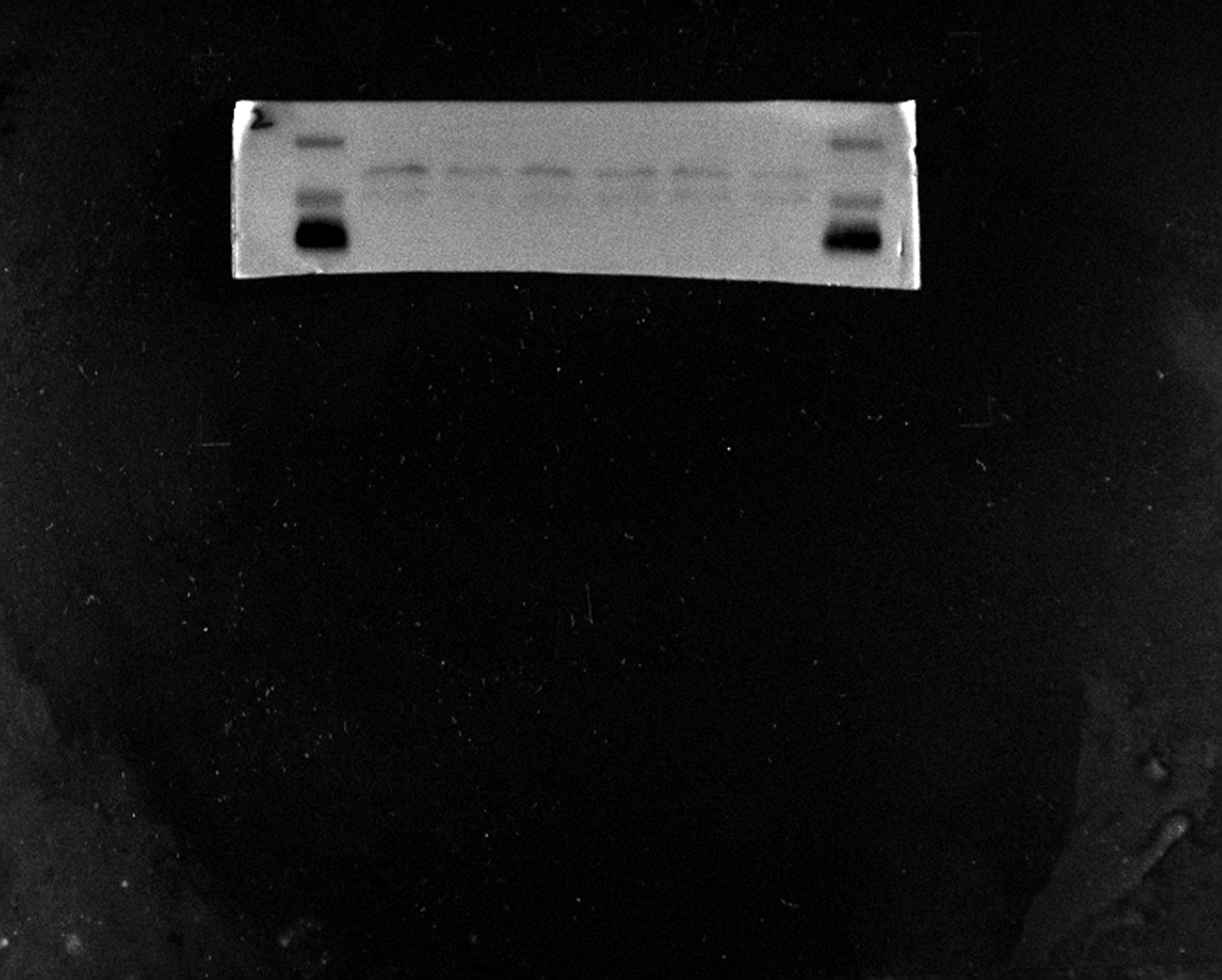

Supplement: Supplementary file 3 [file Data_Sheet_3.ZIP › cells for WB/FIS 3S(25CAI,20-15-10)-HC1.tif]

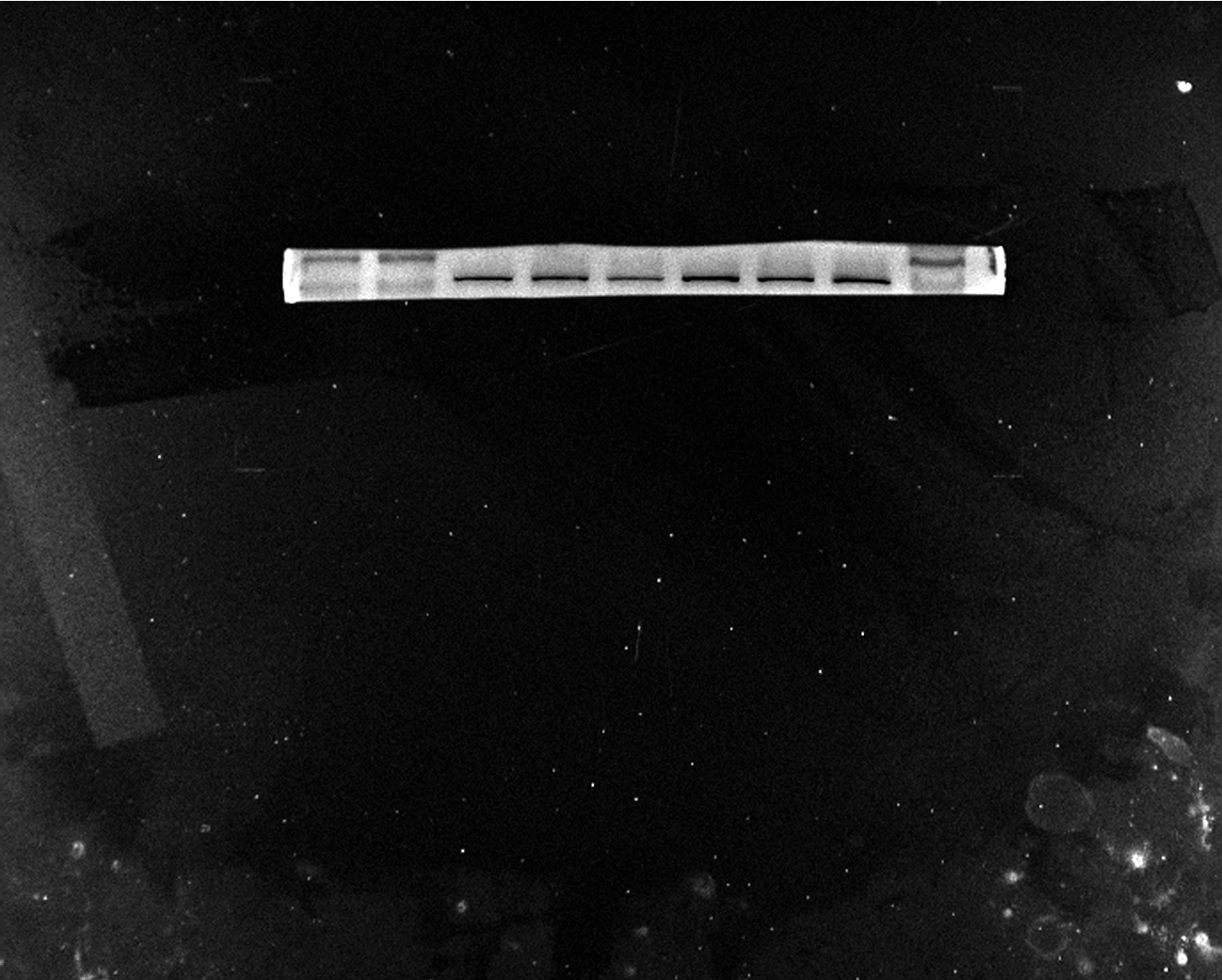

Supplement: Supplementary file 3 [file Data_Sheet_3.ZIP › cells for WB/MFN2 40S(70CAI)-hc.tif]

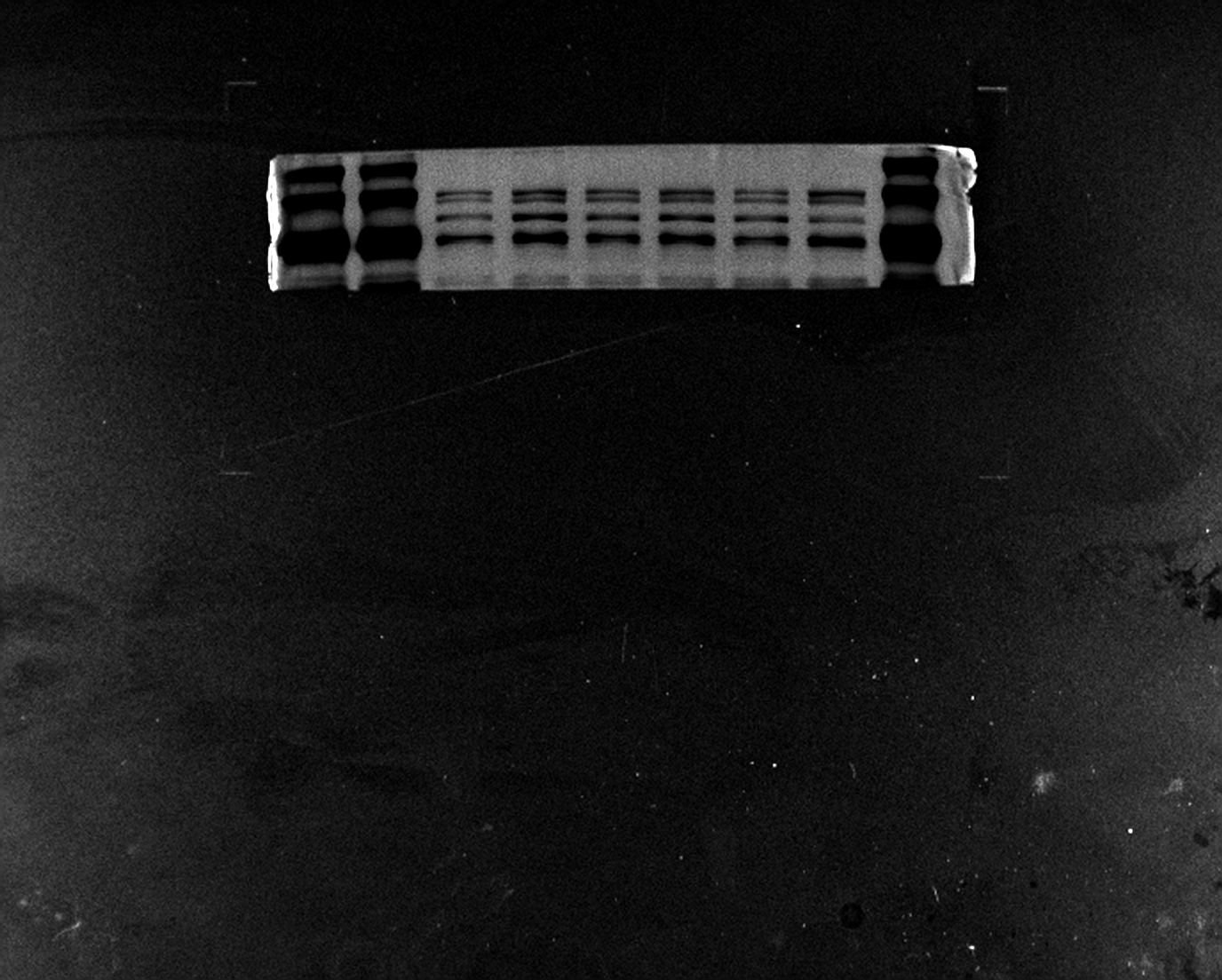

Supplement: Supplementary file 3 [file Data_Sheet_3.ZIP › cells for WB/NRF1 120S(250-100-70-50)-HC.tif]

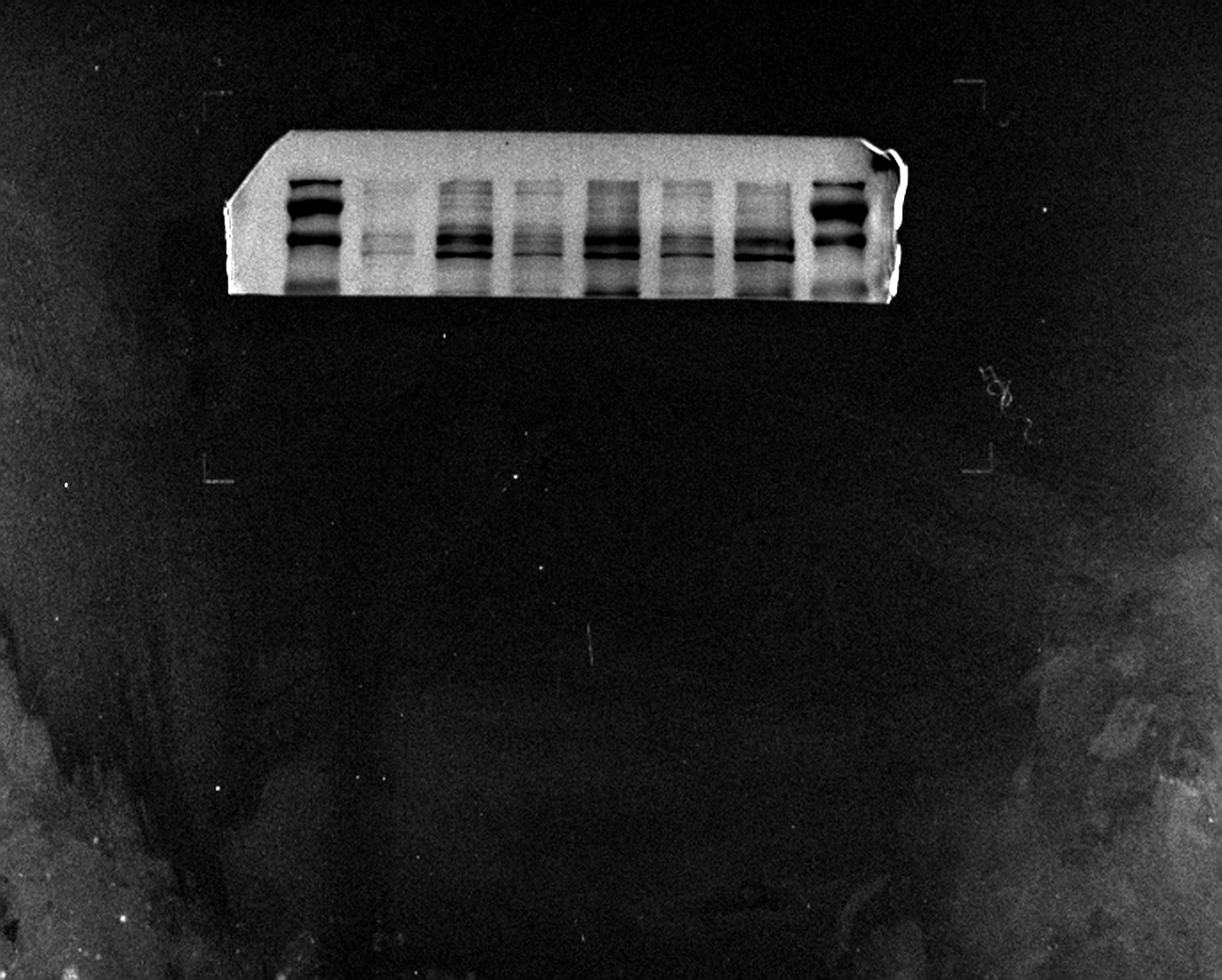

Supplement: Supplementary file 3 [file Data_Sheet_3.ZIP › cells for WB/NRF2 5MIN-HC.tif]

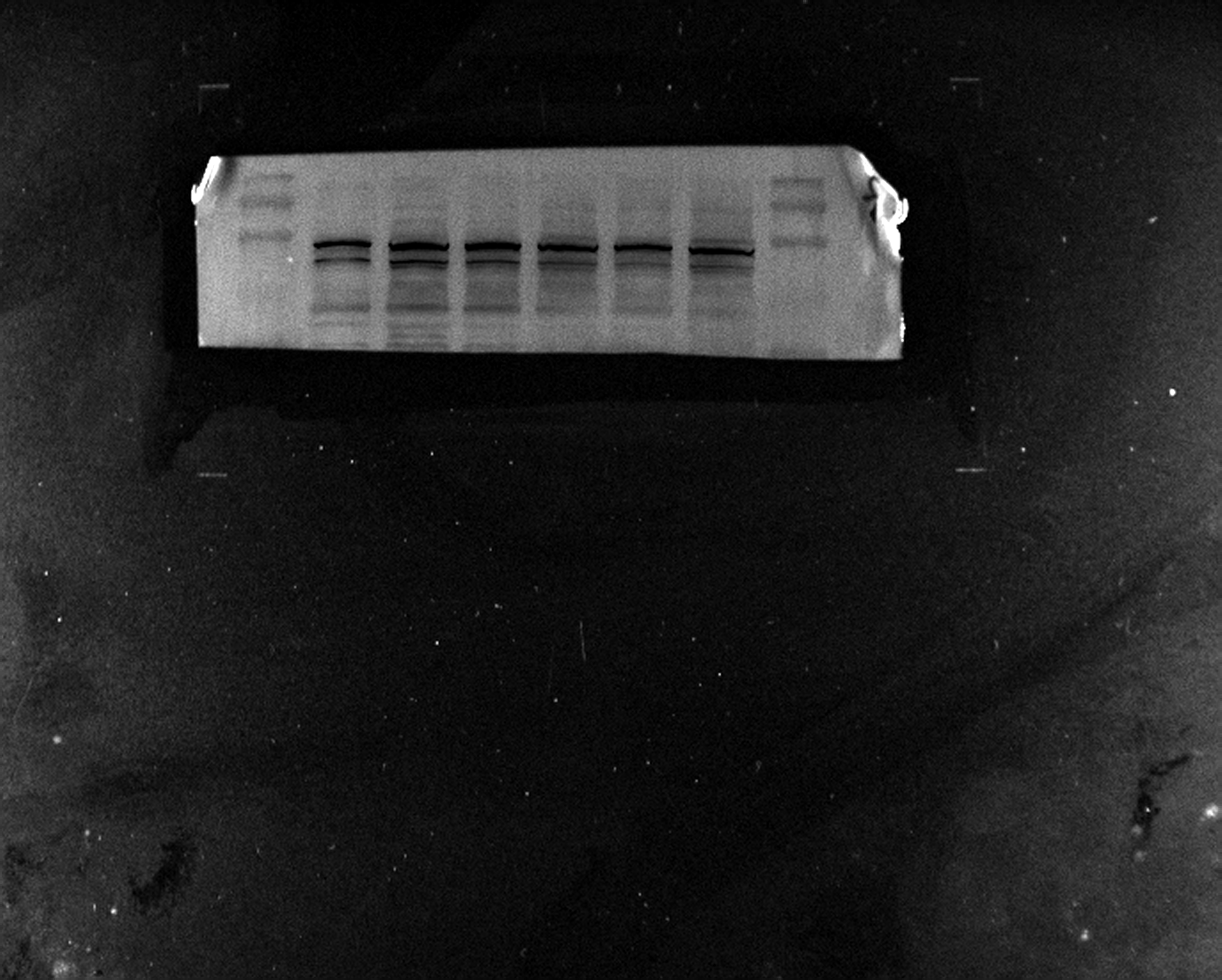

Supplement: Supplementary file 3 [file Data_Sheet_3.ZIP › cells for WB/OPA1(-50) 3MIN-HC.tif]

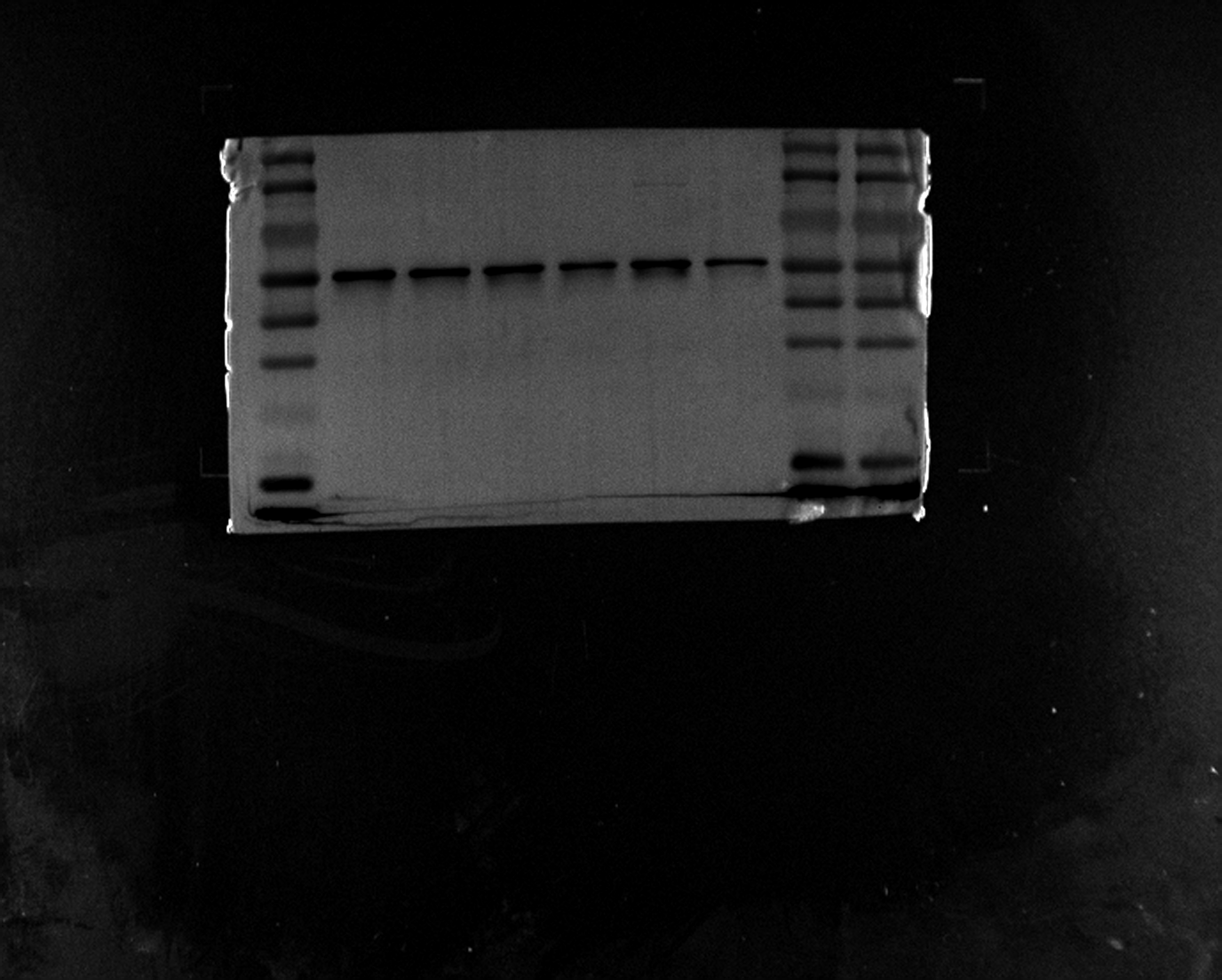

Supplement: Supplementary file 3 [file Data_Sheet_3.ZIP › cells for WB/PAKIN 3MIN-HC.tif]

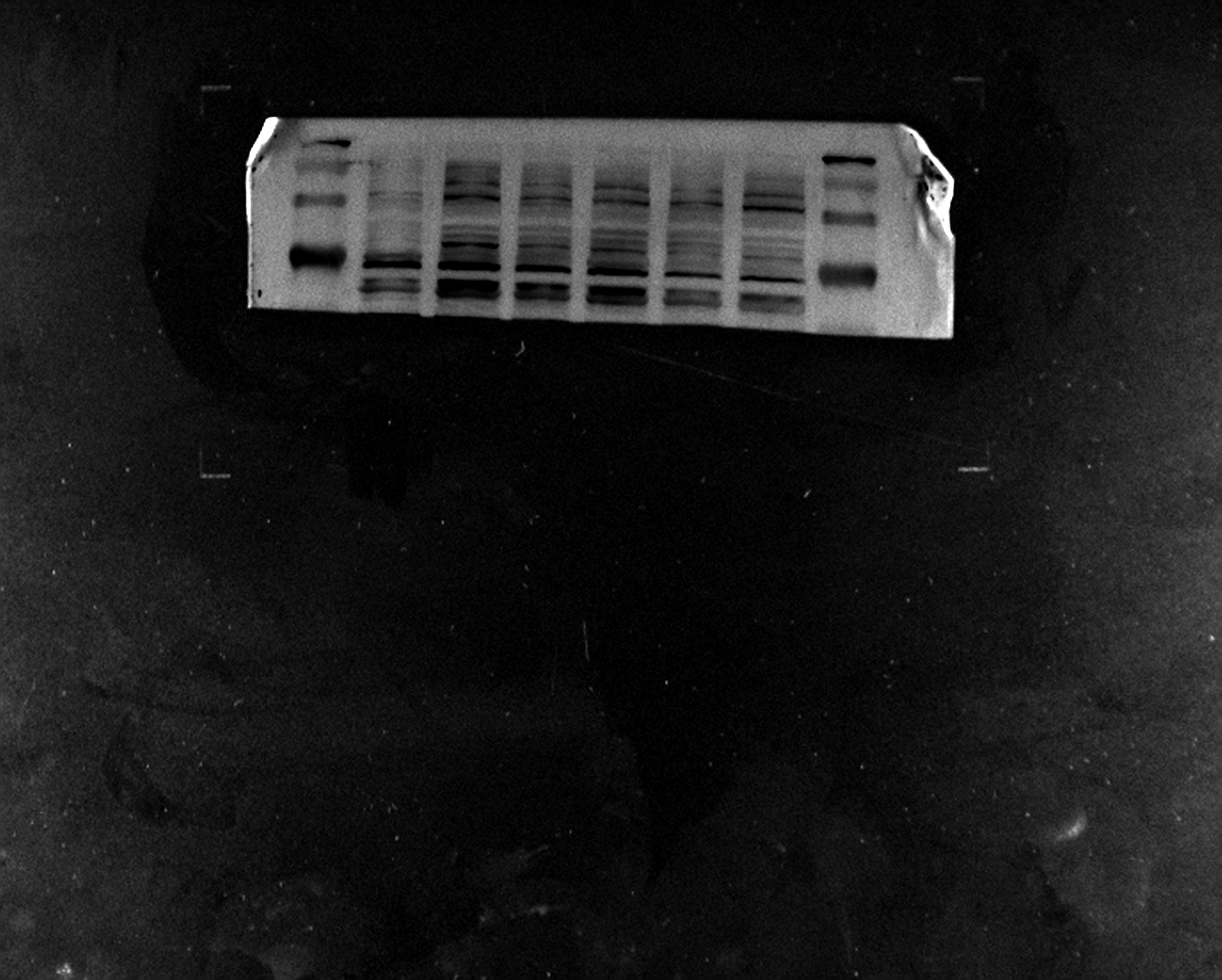

Supplement: Supplementary file 3 [file Data_Sheet_3.ZIP › cells for WB/PGC-1a 2min(250-150-100-70-50)-hc.tif]

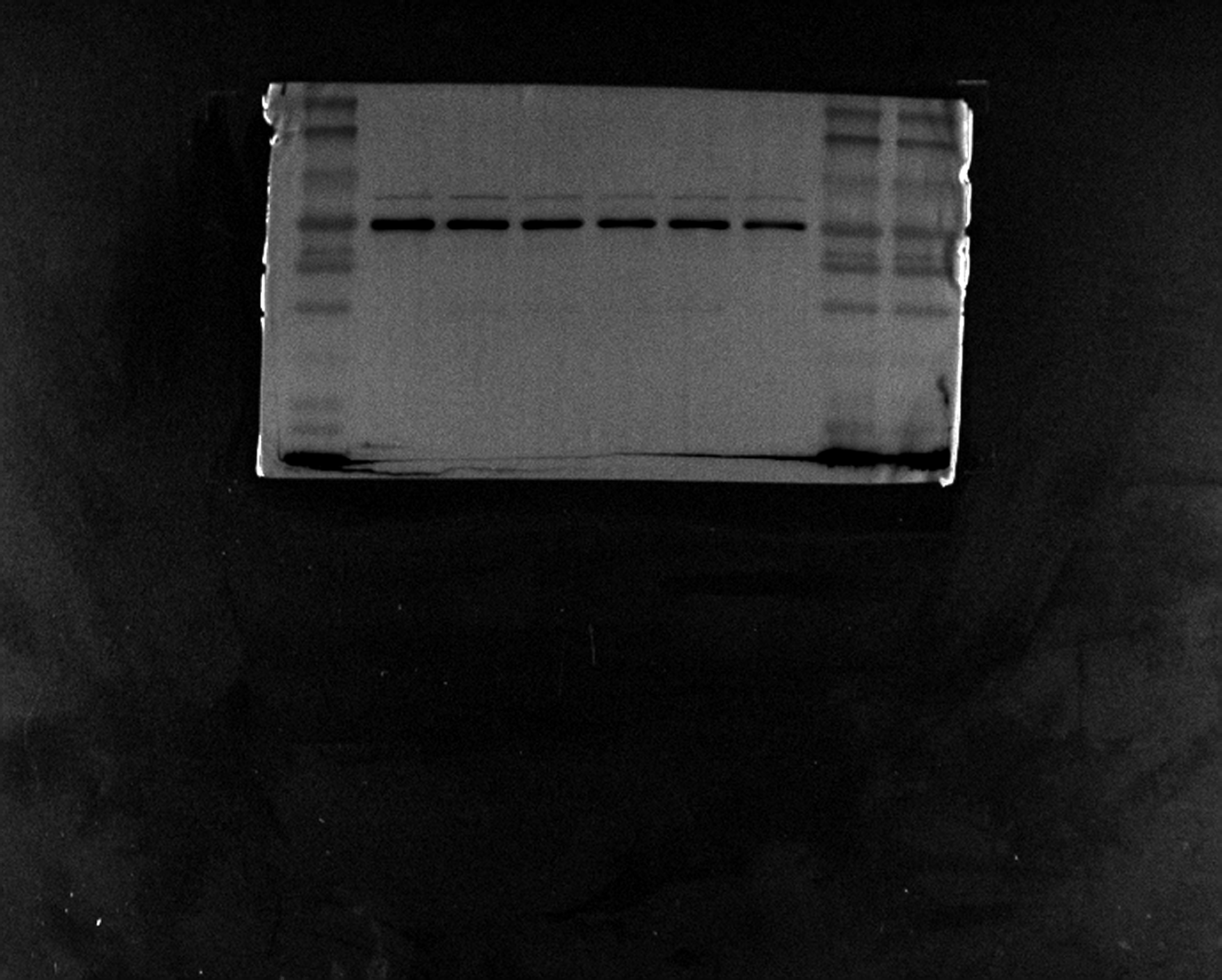

Supplement: Supplementary file 3 [file Data_Sheet_3.ZIP › cells for WB/PINK1 120S-HC.tif]

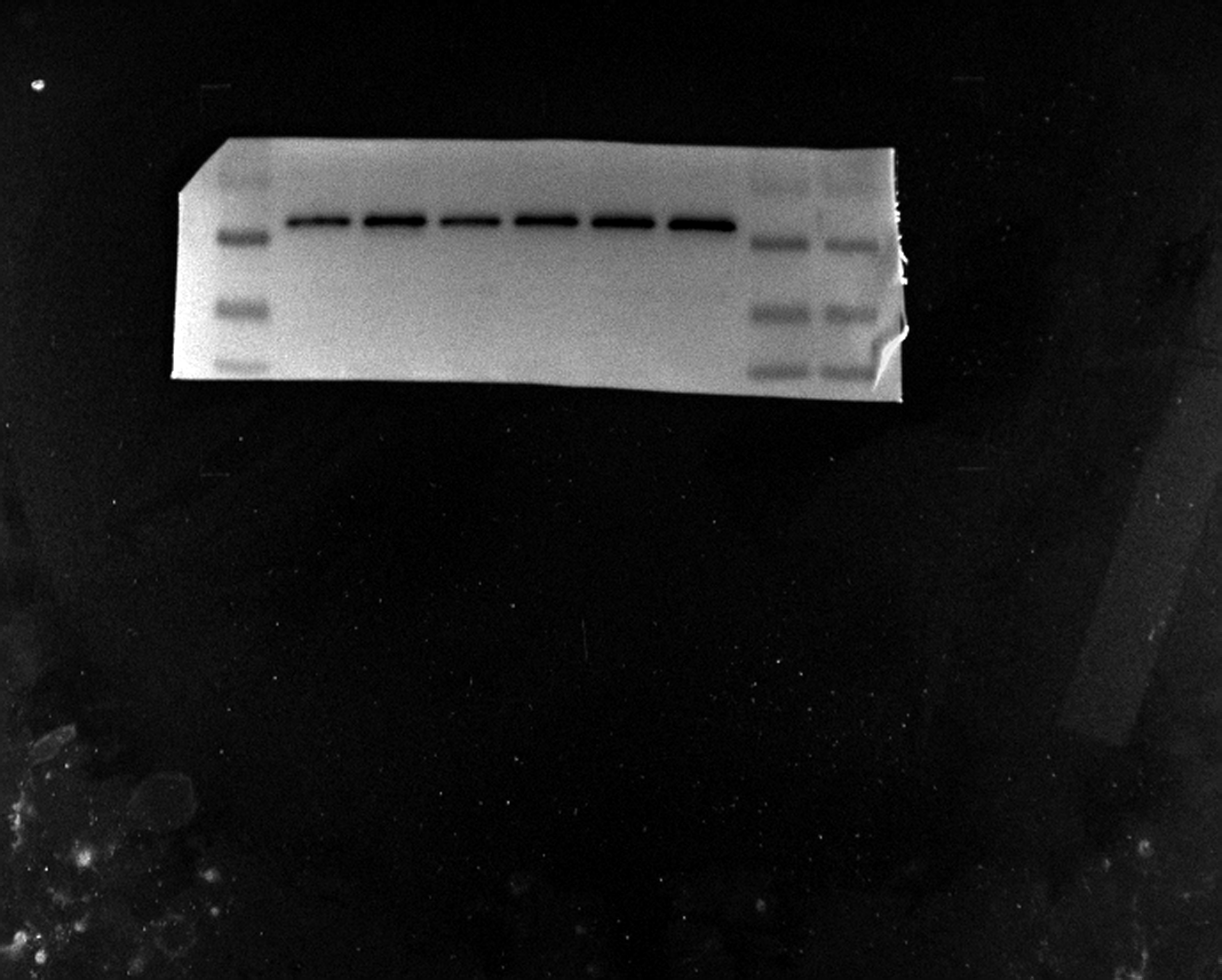

Supplement: Supplementary file 3 [file Data_Sheet_3.ZIP › cells for WB/TFAM 3S-HC.tif]

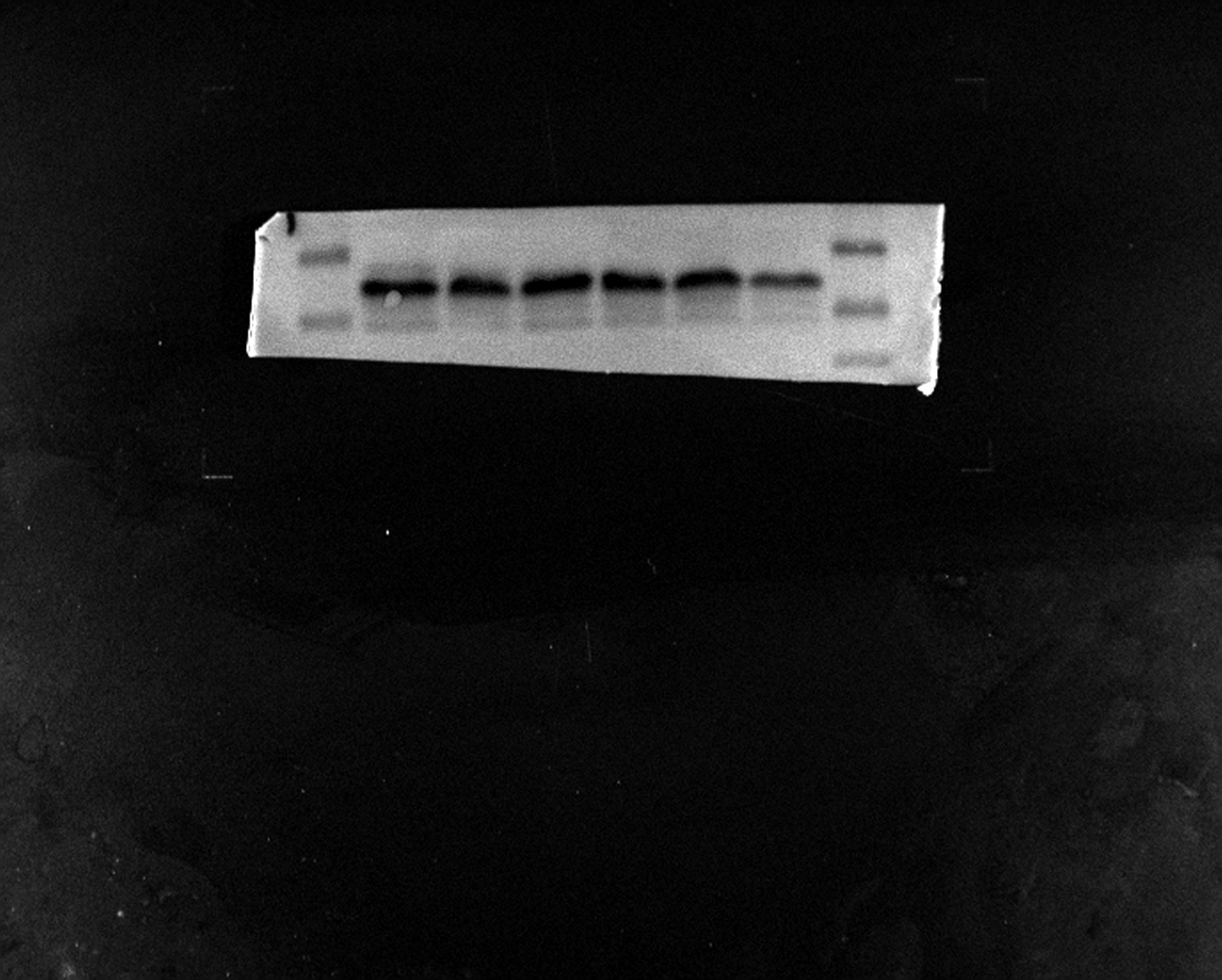

Supplement: Supplementary file 3 [file Data_Sheet_3.ZIP › cells for WB/lc3 3s-hc(25cai,20-10).tif]

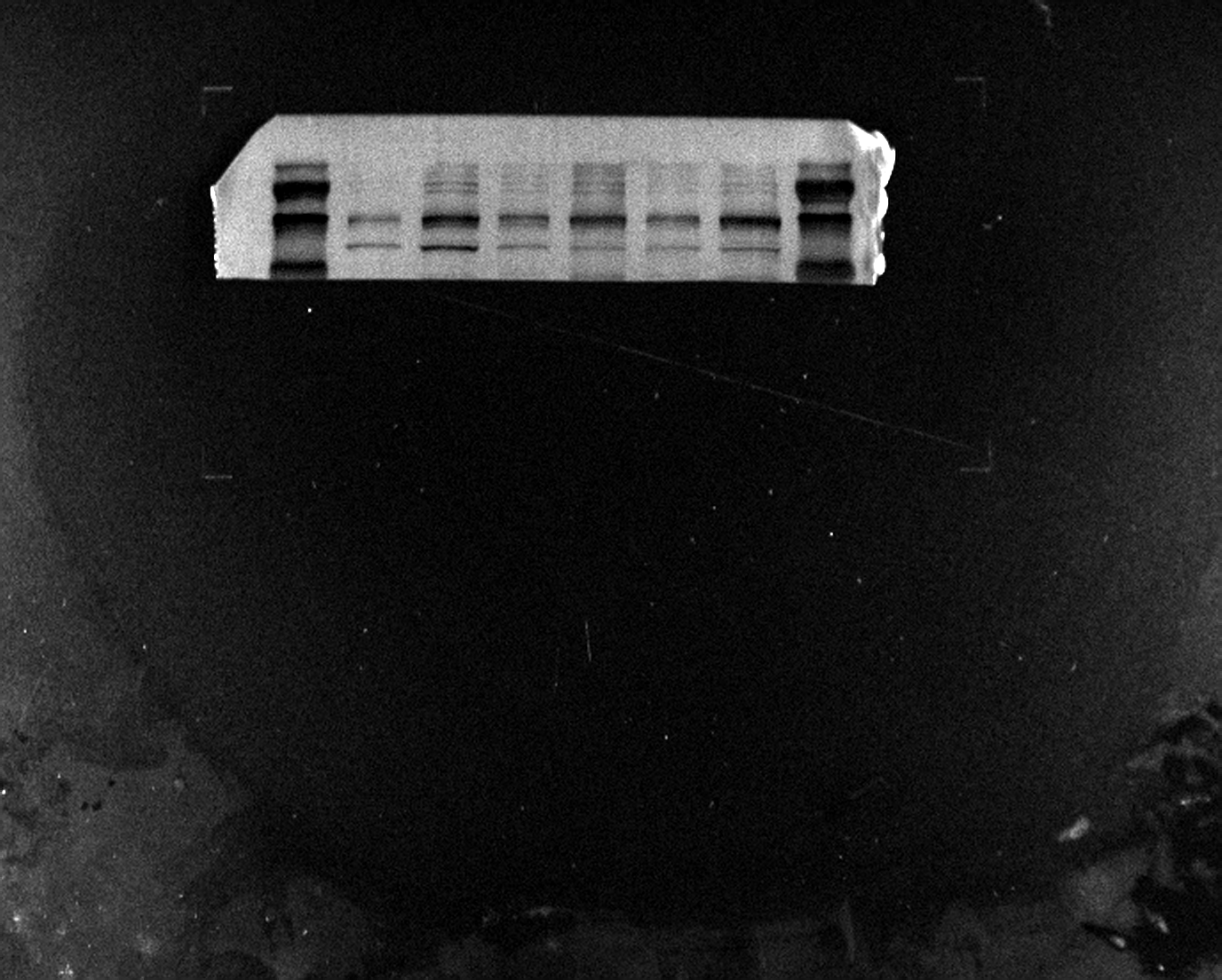

Supplement: Supplementary file 3 [file Data_Sheet_3.ZIP › cells for WB/mfn1 1S-HC.tif]

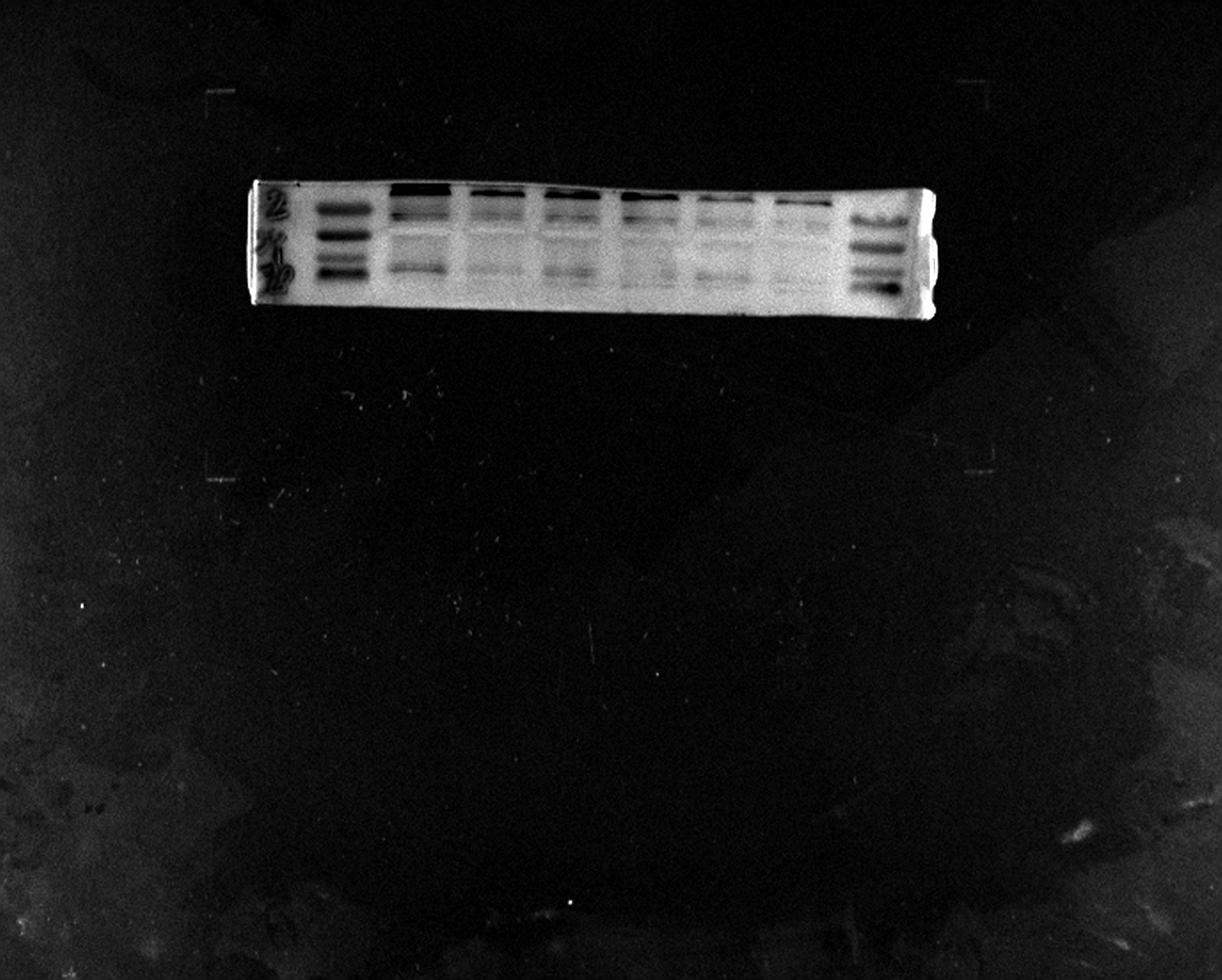

Supplement: Supplementary file 3 [file Data_Sheet_3.ZIP › cells for WB/p62 3min-hc(70-50-40-35-25).tif]
